# Genome-wide analysis uncovers high frequency, strong differential chromosomal interactions and their associated epigenetic patterns in E2-mediated gene regulation

**DOI:** 10.1186/1471-2164-14-70

**Published:** 2013-01-31

**Authors:** Junbai Wang, Xun Lan, Pei-Yin Hsu, Hang-Kai Hsu, Kun Huang, Jeffrey Parvin, Tim H-M Huang, Victor X Jin

**Affiliations:** 1 Department of Pathology, Oslo University Hospital – Norwegian Radium Hospital, Montebello, 0310 Oslo, Norway; 2 Department of Biomedical Informatics, The Ohio State University, 460 W 12th Ave., 212 BRT, Columbus, OH 43210, USA; 3 Department of Molecular Medicine, Institute of Biotechnology, University of Texas Health Science Center, San Antonio, TX, 78245, USA

## Abstract

**Background:**

An emerging Hi-C protocol has the ability to probe three-dimensional (3D) architecture and capture chromatin interactions in a genome-wide scale. It provides informative results to address how chromatin organization changes contribute to disease/tumor occurrence and progression in response to stimulation of environmental chemicals or hormones.

**Results:**

In this study, using MCF7 cells as a model system, we found estrogen stimulation significantly impact chromatin interactions, leading to alteration of gene regulation and the associated histone modification states. Many chromosomal interaction regions at different levels of interaction frequency were identified. In particular, the top 10 hot regions with the highest interaction frequency are enriched with breast cancer specific genes. Furthermore, four types of E2-mediated strong differential (gain- or loss-) chromosomal (intra- or inter-) interactions were classified, in which the number of gain-chromosomal interactions is less than the number of loss-chromosomal interactions upon E2 stimulation. Finally, by integrating with eight histone modification marks, DNA methylation, regulatory elements regions, ERα and Pol-II binding activities, associations between epigenetic patterns and high chromosomal interaction frequency were revealed in E2-mediated gene regulation.

**Conclusions:**

The work provides insight into the effect of chromatin interaction on E2/ERα regulated downstream genes in breast cancer cells.

## Background

An intriguing question in biology is how are genes organized and regulated in the three dimensional space of the nucleus. Transcriptional regulation was thought to be one dimensional along the linear genomic DNA sequence until the wide application of chromatin structure capture experiments, such as Fluorescence In Situ Hybridization (FISH) [1] and Chromatin Conformation Capture (3C) assay [2]. A recent Hi-C protocol, an emerging high throughput technology, has the ability to probe three-dimensional (3D) architecture and capture chromatin interactions in a genome-wide scale [3]. In the study, Lieberman-Aiden et al. modeled the Hi-C data as a probability matrix at a large scale (1 Mb resolution) and revealed the folding principles of genome organization with a sub-domain of chromatin to form genome-wide compartments. However, this study did not quantitatively correlate the 3D chromatin interactions with epigenetic marks, gene expression profiling, and transcriptional regulation. Among the recent efforts to study the effect of genomic chromatin organization on gene regulation, one study [4] showed high correlation between binding sites of CCCTC-binding factor (CTCF) and chromatin interaction identified using the Hi-C data [3]. However, it still lacked in the utilization of other publicly available resources such as epigenetic modifications data. Moreover, the initial single time point Hi-C experiment can only depict a static chromatin structure. Thus, the questions of how the chromatin organization changes upon environmental stimulation such as hormone and chemicals and how these genomic interactions are associated with disease development and progression remains elusive.

In this study, utilizing an estrogen receptor-α (ERα) positive breast cancer cell line, MCF7, before (control) and after estrogen treatment (E2-treated) as a model system, we investigate these biological questions and address how E2 stimulation will affect chromatin interactions resulting in altering gene regulation and their relations with epigenetic modification states. About ~75% breast tumors is in response of estrogen through ERα, which has been reported to regulate distant target genes by long-range chromatin interactions in our previous study [5]. Other previous studies have also linked chromatin organization changes to ERα positive patients and prognoses of the disease [5-11]. Thus, it is reasonable to speculate that these chromatin structure changes contribute to tumor development and proliferation.

To further address this at a genome-wide scale, we performed the Hi-C protocol in MCF7 cells at E2-treated and control conditions. We identified chromosomal interacting regions with different levels of interacting frequency as well as E2-mediated differential chromosomal interactions, and further correlated them with eight histone modification marks, DNA methylation, regulatory elements regions, ERα and Pol-II binding activities. By integrating different ‘omics data in MCF7 cells, we sought to reveal associations between high frequent interaction regions, differential chromosomal interactions, epigenetic modifications and gene regulations upon E2 stimulation, and to provide insight into the effect of chromatin interaction on E2/ERα regulated downstream genes in breast cancer cells.

## Results

### Identification of E2-mediated high frequency chromosomal interacting regions

We applied the Hi-C method [3], an experimental protocol developed for identifying genome-wide high order chromosomal interacting regions, to the E2-treated and control conditions in MCF7 cells. MCF7 is a model breast cancer cell line widely used for mechanistic studying of ERα positive breast cancer. A total of ~111 and ~125 millions of raw paired sequence reads were obtained for E2-treated and control conditions respectively. Of them, ~80 and ~92 millions reads were uniquely mapped to the human genome. We then divided the whole genome into a 1 Mb window-size of chromosome regions, followed by calculating a Z-score (see the Methods) for each chromosome region [12], where a Z-score measures how many standard deviations an observed value is above or below the mean value. This was used to illustrate the relative intensity of chromosomal interactions in a 1 Mb resolution. For example, a higher positive Z-score indicates the observed chromosome region has a much higher interaction frequency than the average. We then constructed genome-wide chromosomal interaction matrices for both E2-treated and control conditions as shown in Additional file 1: Figures S1a,b respectively. We further computed chromosomal interaction frequency for every 1 Mb region based on the Z-score, i.e. the number of non-zero Z-scores in the region divided by the total number of 1 Mb regions in the human genome (Table 1). We found that under the same cutoff value for chromosomal interaction frequency, the number of interactions at E2-treated condition was less than that in the control condition. In particular, the difference was more dramatic at a higher interaction frequency (e.g., > = 10%) than that at the lower one. The same trend was also observed in a repeated analysis with 2 Mb window-sizes, Additional file 1: Tables S1.

**Table 1:** Distribution of chromosomal interaction frequency in the human genome (1 Mb resolution), where the number of regions with interaction frequency greater than and equal to 1%, 5%, 10%, 20%, 30%, 40%, 50% and 60% are shown respectively

**Chromosomal Interaction frequency ( >=)**	**Number of regions in control condition**	**Number of regions in E2-treated condition**
1%	2851	2845
5%	2061	1581
10%	234	129
20%	15	12
30%	10	7
40%	3	1
50%	1	0
60%	0	0

**Figure 1:**
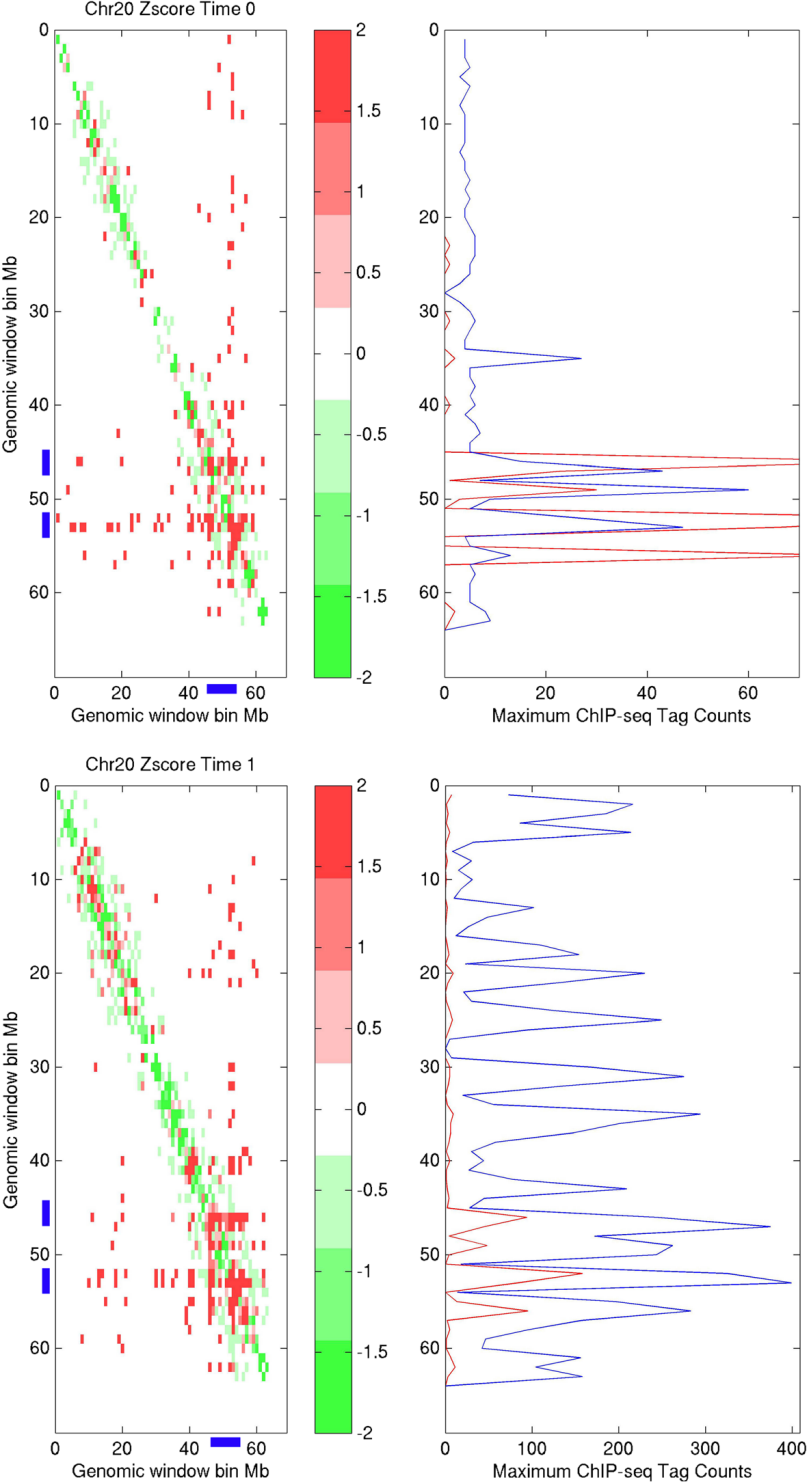
**Chromosomal interaction hotspots. **Upper panel: intra-chromosomal interaction for chromosome 20 at control condition; down panel: intra-chromosomal interaction for chromosome 20 at E2-treated condition; right panel, red smooth line represents detected number of ERα binding sites in the region (1 Mb resolution), and blue smooth line is the maximum read counts in the region (1 Mb resolution); left panel, chromosomal interaction hot regions (within amplified regions) that identified by this study are colored by blue bar at X-axis and Y-axis, respectively; positive and negative Z-scores are colored by red and green color, which indicate the observed chromosome region has higher and lower interaction frequency than the average, respectively.

We subsequently examined the top 10 highest chromosomal interacting regions above 30% interaction frequency at control condition (Table 2), as defined as top 10 hot regions, where 7 of them remain above 30% interaction frequency and the rest 3 drop to > =25% interaction frequency at E2-treated condition. Although the difference of interaction frequencies between the E2-treated and control conditions is not significant, all chromosomal regions showed higher interaction frequency at the control condition than those at the E2-treated condition, suggesting that the chromosomal interaction frequency may be reduced upon E2 stimulation. To understand the ERα binding information within these high frequency interacting regions, a publicly available [13] ChIP-seq data of ERα at the E2-treated and control condition in MCF7 cells was used. A total number of ~1,000 and ~12,047 peaks were called by using W-ChIPeaks [14] in control and an E2 treated dataset, respectively. These peaks were used to examine the relationship between ER occupancy and interaction frequency. The number of ERα binding sites was greatly increased in the E2-treated condition in the top 10 interaction hot regions (for instance, ~160 in E2-treated condition verses ~30 in the control condition for chr20:52000001:53000000; Table 2). In particular, the results showed a significant increase in binding intensity of ERα in E2-treated condition when compared to that in the control condition (i.e. ~400 in E2-treated condition verse ~50 in the control condition for chr20:52000001:53000000; Table 2). Genome-wide, the regions with higher interaction frequencies also had higher ERα binding occupancy: for example, at chromosome 3, 17 and 20 (Figure 1 and Additional file 1: Figures S2, 3).

**Table 2:** Chromosomal regions (1 Mb resolution) with highest interaction frequencies (> = 30% in control condition; T0)

**Chromosome regions (interaction frequency > =30%)**	**T0 ChIP-seq maximum read count**	**T0 ChIP-seq binding sites**	**T0 interaction frequency%**	**T1 ChIP-seq maximum read count**	**T1 ChIP-seq binding sites**	**T1 interaction frequency%**
chr3 62000001 63000000	6	0	31.4955	89	8	28.5573
chr3 63000001 64000000	16	5	32.1558	64	21	27.5008
chr3 64000001 65000000	8	8	44.5031	105	65	37.141
chr17 54000001 55000000	114	7	30.6042	308	46	25.7181
chr17 55000001 56000000	14	14	35.8864	95	62	31.9247
chr17 56000001 57000000	15	47	37.7022	110	132	32.4199
chr17 57000001 58000000	10	6	39.7161	169	36	34.2357
chr20 45000001 46000000	15	60	37.5041	244	148	31.4955
chr20 51000001 52000000	26	69	43.5787	325	182	36.4807
chr20 52000001 53000000	47	34	53.549	399	167	45.2955

The corresponding interaction frequency in E2-treated condition (T1) is also listed. ChIP-seq read count represents ERα binding intensity, and ChIP-seq binding sites represent the number of binding sites.

In a repeat analysis using 2 Mb window-sizes, similar results were obtained (Additional file 1: Figures S4, 5, 6). This suggests that E2 may have a higher chance to trigger stronger ERα binding and regulate genes at the higher interaction frequency regions. As a result, three loci close to the top 10 interaction hot regions were selected, including THRAP1 (17q23), INTS2 (17q23) and CADPS (3p14), genes within a higher interaction frequency region (i.e. chromosomal interaction frequency >30% in E2 treated condition), and C16orf65 (16p12) from a middle level interaction frequency region (i.e. chromosomal interaction frequency ~7% in E2 treated condition), and ZIM2 (19q13) as a negative control (i.e. chromosomal interaction frequency ~3% at E2 treated condition), to validate the E2-treated chromosomal interactions by quantitative 3C-PCR (3C-qPCR). The interaction was observed in THRAP1, INTS2, CADPS and C16orf65 loci upon 1 hr of E2 treatment (Additional file 1: Figure S7).

### Linking chromatin interaction with genome rearrangement

Cancer cells are highly disrupted. For MCF7 cells, a previous study [13] has shown that a total of 314 (157 pairs) break-points were induced by somatic rearrangement using BACs combing with pyrosequencing techniques. Interestingly, we observed a high overlap rate between the hot 10 regions and genomic break and fusion sites. By comparing a total of 314 break-points, 69 of 314 (22%) are within the top 10 hot regions identified in our study (*P* < 1E-20) while only 1 is within the top 10 cold regions. This may suggest interaction hot regions on chromosome 3, 17, 20 are frequently rearranged in MCF7 cells. However, a primary concern of identifying chromatin interaction is that fusion sites produced by genomic rearrangement might introduce false positive interactions between two physically joint loci. Several lines of evidence may ease this concern. First, one advantage of the Hi-C protocol is that the hybrid junctions are labeled with biotin; therefore, if a junction was caused by a rearrangement, no biotin label is added and it cannot be pulled down by avidin. Thus, theoretically, Hi-C protocol can eliminate these types of artificial interactions due to the genomic rearrangements. Second, in a recent study in K562 cells [15], no interaction identified using Hi-C data was found within 20 kb distance of 25 identified fusion sites. Third, another recent study demonstrated that three-dimensional organization of genome contributes to chromosomal translocations in mouse leukemia virus (A-MuLV) which transformed pro-B cells by combining Hi-C and high-throughput genome-wide translocation sequencing (“HTGTS”) analysis [16]. The same study found that intra-chromosomal translocation frequency is correlated with Hi-C contact probability.

Although we did not perform HTGTS in this study, nevertheless, our simple comparison between fusion sites and interacting regions suggests that the highest frequency interactions may facilitate the chromosome translocation in this breast cancer cell line. We further speculate that chromosome translocation may further enhance the chromatin reorganization at the junction region and promote the formation of chromatin interaction. Such an intensive interactive environment may increase the chance of aberrant DNA amplification, and thus form multiple copy number inverted repeats or tandem repeat regions on these chromosomes. The inter-chromosomal interactions identified between these fusion sites may represent actual intra-chromosomal interactions on the rearranged derivative chromosome in the cancer cell. Taken together, our initial results suggest a link between chromosomal interactions with translocations in the breast cancer model system.

### Characterization of E2/ERα-regulated genes in both highest and lowest frequency chromosomal interacting regions

In the top 10 hot regions (i.e., highest frequency chromosomal interacting regions), there are 69 E2/ERα-regulated genes. Functional gene ontology (GO) analysis (Table 3) shows that the top GO biological processes are protein amino acid dephosphorylation and intracellular receptor-mediator signaling pathways. Tissue expression study of those genes suggests that they are highly enriched in breast (mammary gland) cancer disease, mammary gland normal, mammary gland neoplasia and ovary neoplasia. More importantly, those genes are often linked to human disease such as breast cancer and adult human height. A more detailed description of functional annotation results are listed in Additional file 2. Correlation coefficient of expression (~54 genes) profiles [17] between the E2-treated and control conditions is ~0.99, which suggests that E2 stimulation may not alter expression levels of genes in higher frequency chromosomal interaction regions dramatically (Additional file 1: Figure S8). Taken together, the identified 10 hot regions (i.e. interaction frequency > =30%; Table 2) contain many genes that are linked to human disease such as breast cancer, which indicates that chromosomal regions with higher interaction frequency may play a pivotal role in genome regulation.

**Table 3:** Functional annotation of genes located in the top 10 cold (1 in chr5, 5 in chr9, 1 in chr13, 1 in chr15, 1 in chr18 and 1 in chr22) and the top 10 hot (3 in chr3, 4 in chr17 and 3 in chr20) chromosomal interaction regions by using DAVID

	**GO term**	**Tissue expression**	**Disease**	**Pathways**
Top 10 cold regions (45 genes)	phosphatidylcholine biosynthetic process (2 genes); phosphatidylcholine metabolic process (2 genes); ethanolamine and derivative metabolic process (2 genes); intracellular organelle lumen (5 genes); glycerophospholipid biosynthetic process (2 genes).	Thalamus_3rd (7 genes); testis_normal_3rd (14 genes); brain_normal_3rd (3 genes); Cardiac Myocytes_3rd (10 genes); Testis Seminiferous Tubule_3rd (6 genes).	NA	NA
Top 10 hot regions (69 genes)	protein amino acid dephosphorylation (4 genes); dephosphorylation (4 genes); phosphoprotein phosphatase activity (4 genes); intracellular receptor-mediated signaling pathway (3 genes); clathrin coat of trans-Golgi network vesicle (2 genes).	breast (mammary gland) cancer_disease_3^rd ^(32 genes); mammary gland_normal_3rd (33genes); mammary gland_neoplasia_3rd (6 genes); ovary_neoplasia_3^rd ^(3 genes); placenta_normal_3^rd^ (4 genes).	Many sequence variants affecting diversity of adult human height (4 genes); breast cancer (4 genes); CANCER (5 genes).	REACTOME_PATHWAY: Membrane Trafficking (2 genes)

The top 5 of each functional annotation are presented here.

For comparison purposes with the top 10 hot regions, the top 10 cold regions with the lowest interaction frequency was also studied (i.e. interaction frequency < =0.5% at control condition). There are 45 genes in the top 10 cold regions (Additional file 3). The result of functional annotation GO [18] analysis reveals that genes located in cold regions are enriched in phosphatidylcholine biosynthetic process, phosphatidylcholine metabolic process, ethanolamine and derivate metabolic process. For tissue expression tests [18], genes of interaction cold regions are often linked to thalamus, testis, brain and cardiac myocytes (Table 3). However, genes of hot regions are linked to breast cancer disease such as BACH1, RAD51C, CYP24A1 and NCOA3. Such functional difference between genes located in the chromosomal interaction hot regions and cold regions is also observed in the top 50 interaction hot (280 genes) and cold regions (500 genes), respectively (Additional file 1 Table S2; Additional file 4 and Additional file 5). In particular, there are various transcription factor binding sites detected in the chromosomal interaction hot regions but none of them appeared in the interaction cold regions. Thus, the results suggest that estrogen may stimulate those mammary-specific genes of higher frequency interaction with other genomic regions to drive the breast tumorigenisis.

Next, we examined whether these breast cancer related genes were associated with genomic break and fusions. Interestingly, we found that two of five genes, namely RAD51C and NOCA3, have DNA break sites within the gene body region. In contrast to the well-known chromosome 9 and 22 fusion (Philadelphia chromosome) in human chronic myelogenous leukemia or the translocation of ETS family transcription factors in prostate cancer, no dominant causal gene fusion events in breast cancer patients has yet been reported. The difference between these cancers combined with the observation that breast cancer genes are enriched within chromatin interaction hot regions which has a high rearrangement rate suggests a critical role of chromatin interactions in breast cancer. This result indicates another potential pathway for breast cancer development, in which, oncogenes might be brought to an intensive interacting environment such as these hot regions, thus more subject to the chromosome translocation.

Next, the selected top 10 cold and hot regions were compared to a previous study conducted by the ChIA-PET in MCF7 cells [19], a different technology to identify E2/ERα regulated chromatin interactions. Interestingly, the identified top 10 hot regions in this study are overlapping with interaction regions identified by ChIA-PET experiments (Additional file 6). However, for the top 10 interaction cold regions, none of them are matched to the interaction regions provided by Fullwood et al. This comparison not only validated our technology and analysis but also further supported a notion that highly frequent interaction regions such as the top 10 hot interaction regions truly exist. The high interaction regions may drive breast cancer development and progression.

### Quantification of E2-mediated differential chromosomal interactions

**Figure 2:**
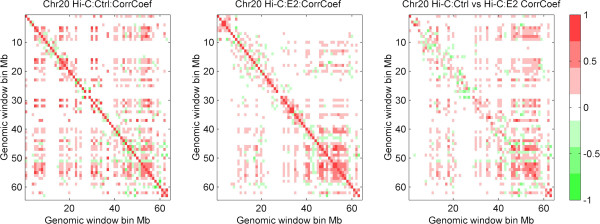
**Correlation coefficient matrices of intra-chromosomal interactions. **Left, middle and right panel represent colored heat map of correlation coefficient matrices of chromosome 20 intra-chromosomal interactions (1 Mb resolution) in control condition, E2-treated condition, and between control and E2-treated conditions, respectively. Here positive and negative correlation coefficients are color scaled to red and green, respectively.

**Figure 3:**
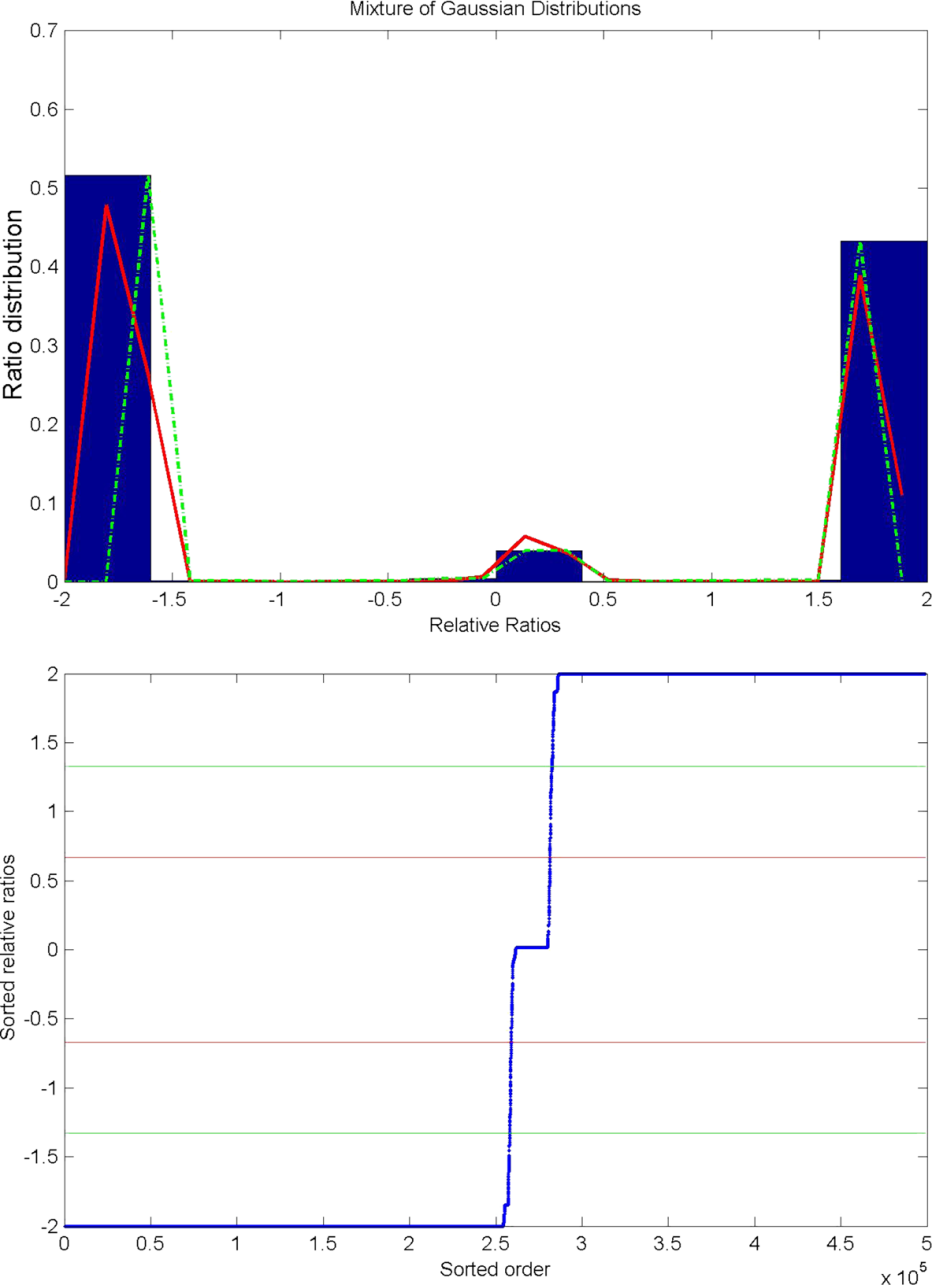
**Distribution of relative ratios of chromosome interaction changes. **Upper panel: Histogram of relative ratios (chromosomal interaction changes in 1 Mb resolution, E2-treated *vs *control condition). Lower panel: sorted relative ratios, red smooth line is relative ratio equals 0.67 (e.g., a 2 fold change) and green smooth line is relative ratio equals 1.33 (e.g., a 5 fold change). Non-interaction elements are excluded from analysis such as Z-score equals 0 in both control and E2-treated interaction matrices. A 10-fold interaction change is expected when the relative ratio equals 1.63, gain and lost interactions are equivalent to the relative ratio 2 and −2, respectively.

After revealing the correlation between chromosomal interaction frequency and gene regulations such as gene expression and epigenetic modifications (Additional file 1: Figures S9 and S10) in E2-treated and control conditions, respectively, we wanted to further quantitatively measure the difference of chromosomal interactions between the E2-treated and control conditions. First, we plotted the differential chromosomal interactions heatmap comparing the overall interaction frequency changes before and after E2 treatment. The results suggest that the overall chromosome interaction frequency and pattern are changed for all chromosomes (i.e. Figure 2, chromosome 20, the intra-chromosomal interaction frequency and pattern are clearly changed before and after E2 treatment). Then, by calculating a relative ratio (see Methods section) for each pair of interacting 1 Mb window-size regions between the two conditions, a human chromosomal interaction change could be estimated. The distribution of the relative ratios for all regions (Figure 3) showed that a majority (~94%) of absolute relative ratios close to 2 (i.e. a greater than 10-fold change of interaction strength between the two conditions). A similar pattern was also observed in 2 Mb window-size (Additional file 1: Figures S11, 12). This suggests that most of chromosomal interactions are either strengthened or weakened after E2-treatment.

An interaction change with an absolute relative ratio of 2 and the corresponding Z-scores of larger than 1 is defined as a strong chromosomal interaction change between the E2-treated and control conditions (Additional file 1: Table S3). This can be further classified into four types, the gain- or loss-, intra- or inter-chromosomal interactions. A detailed list of the four types of interactions is shown in Additional file 7. We observed that there are more than three times the number of gain-intra-chromosomal interactions than the number of loss-intra-chromosomal interactions at E2-treated condition and, by contrast, the number of gain-inter-chromosomal interactions are much less than the number of loss-inter-chromosomal interactions under the same condition. In total, the number of gain-chromosomal interactions is less than the number of loss-chromosomal interactions at E2-treated condition. A similar trend was also observed in the analysis of a dataset with a smaller chromosomal interaction change (i.e. with the absolute relative ratio > = 0.67 and Z-score is not zero; Additional file 1: Table S4).

**Figure 4:**
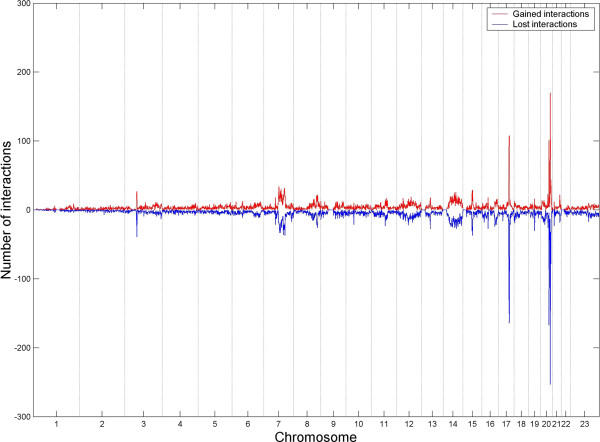
**Dynamical changes of chromosomal interactions between control and E2-treated conditions. **Number of gained (i.e. red smooth line, positive value) and lost (i.e. blue smooth line, negative value) interactions between control and E2-treatd conditions are calculated for every 1 Mb region of human genome based on the four types of the strongest chromosomal interactions (i.e. strong differential gain or loss chromosomal intra or inter interactions, Additional file 7).

**Figure 5:**
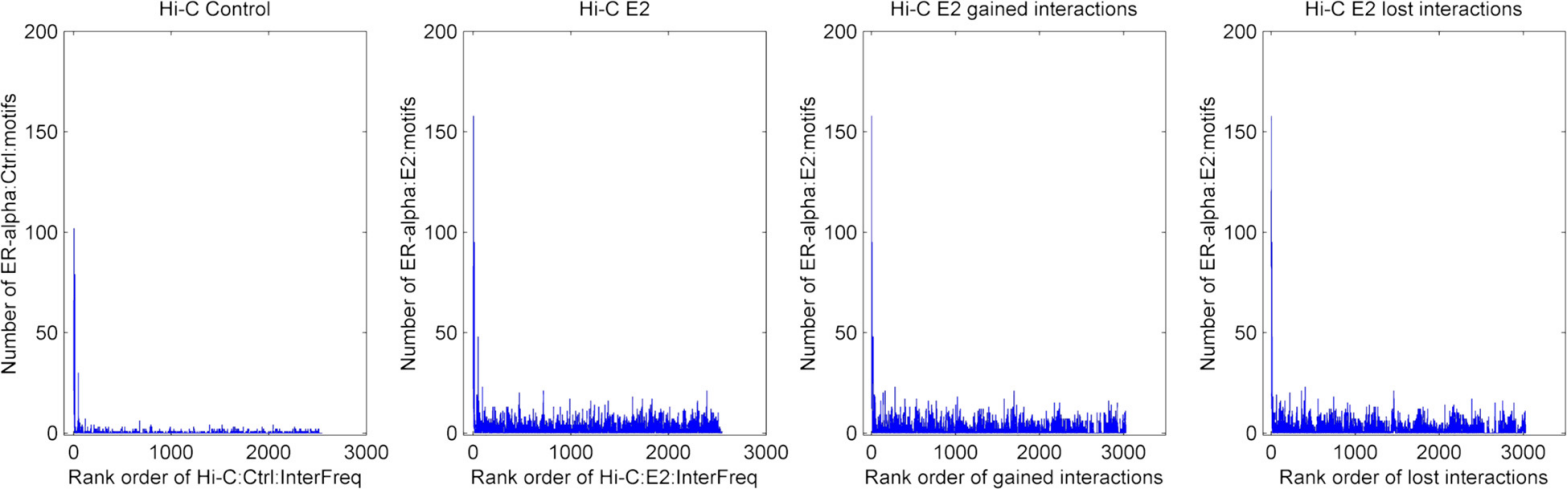
**Association of chromosomal interaction frequency and ER-alpha binding motifs. **ER-alpha binding motifs are identified by applying peak calling program wBELT on public available ER-alpha Chip-Seq experiments. Then the number of ER-alpha binding motifs in every 1 Mb region of human genome is plotted against to the rank order of local chromosomal interaction frequencies in Hi-C experiments. Figures from the left to the right represent such plot in Hi-C control, E2-treated, E2-treated gained interaction only and E2-treated lost interaction only conditions, respectively.

A genome-wide plot of the distribution of strong chromosomal interaction changes along 23 human chromosomes (Figure 4) showed that that the frequency of gain- and loss- interactions in every region (1 Mb resolution) is symmetrically distributed along the chromosomes. A repeated analysis in 2 Mb resolution showed similar results (Additional file 1: Figures S11, 12). Additionally, a scatter plot of the rank order of the number of gain or loss chromosomal interactions verses the number of putative ERα binding sites for 3,029 chromosome regions in human genome was made (Figure 5), which showed the higher interaction frequency and the more ERα binding sites are found. In particular, we observed that seven of the top 10 regions with the most gain- or loss interactions (Additional file 1: Table S5) are overlapping with the top 10 hot regions (Table 2). Together, our results demonstrated that genome-wide chromosomal interactions are changed before and after E2 treatment. For example, inter-chromosomal interactions were gained but higher chromosomal interaction frequency was reduced upon estrogen stimulation. More importantly, chromosomal interaction frequency is positively associated with the number of ERα binding sites.

### Correlation between E2-mediated differential chromosomal interactions and epigenetic modifications

To determine correlations among chromosomal interaction frequency, epigenetic marks and transcriptional regulation, eight publicly available histone marks [20] (H3K4me1, H3K4me2, HK4me3, H3K9me2, H3K9me3, H3K27me3, H3K9ac, H3K14ac), DNA methylation, Pol-II level and regulatory activity (FAIRE) were used to calculate the log transformation of read counts for genes within every 1 Mb window-size region. Here ~16% of 1 Mb chromosome regions (480) were excluded from the analysis because there is no gene in these regions. In order to determine the role of each specific regulatory region for any given gene, we further divided each gene into the three regulatory regions in reference to a 5’ transcription start site (5TSS), 5 Kb upstream, 5 Kb downstream and gene body. Then, the mean of log transformed read counts for each part at the 1 Mb chromosome region were computed and displayed in a heat map (Additional file 8), with the order of chromosome regions sorted by the interaction frequency at the control condition. The results showed that there is a clear separation between the interaction hot regions (regions with the highest chromosomal interaction frequency; lowest panel of Additional file 8; Additional file 1: Figure S9) and the interaction cold regions (regions with the lowest chromosomal interaction frequency; top panel of Additional file 8; Additional file 1: Figure S9).

**Figure 6:**
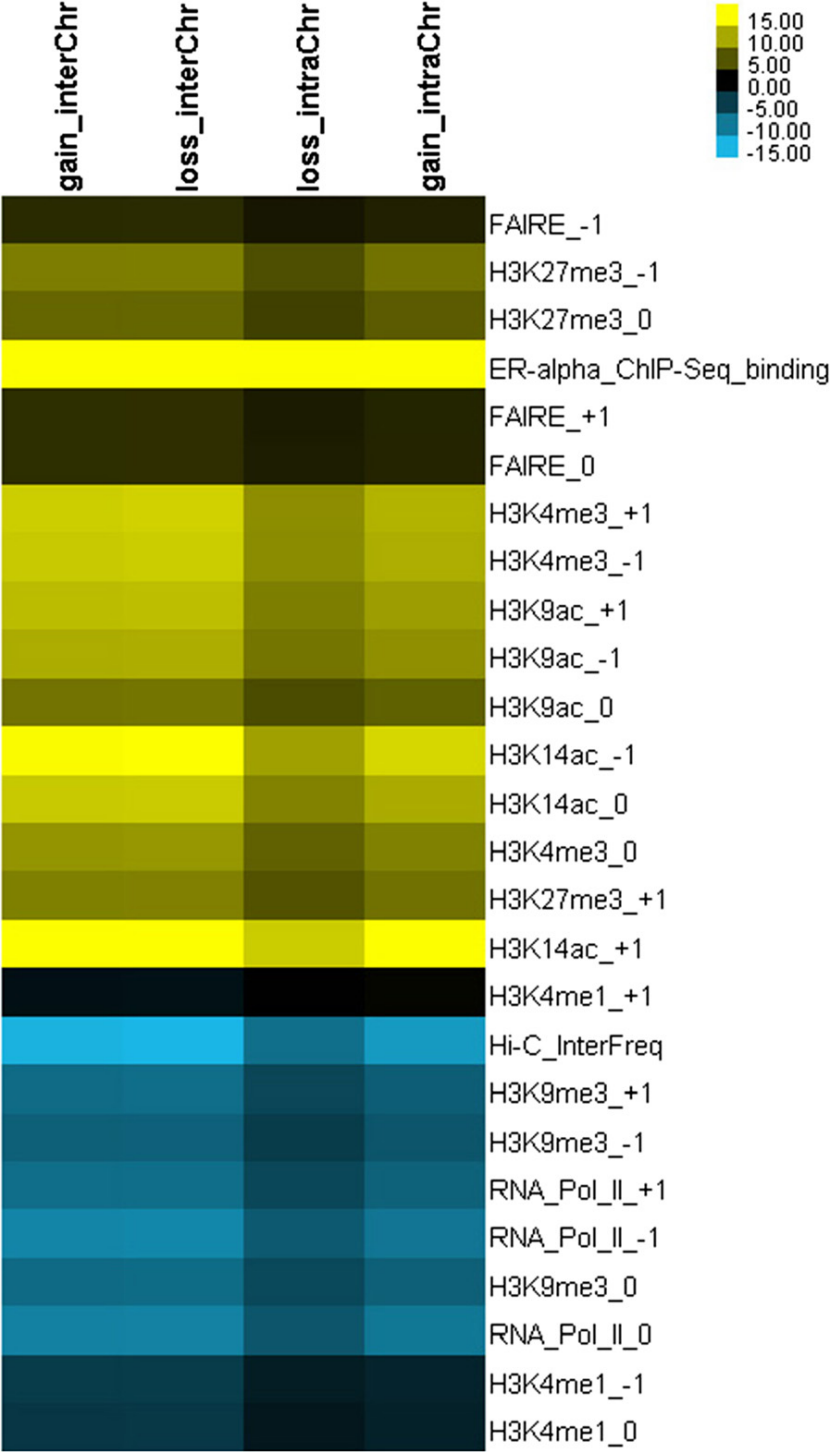
**A heat map of change of histone modifications between E2-treated and control conditions for four types of chromosomal interactions. **Here T-values are obtained by perform *t*-test for genes that were chosen by the identified four types of chromosomal interaction changes (e.g., gain strong inter-chromosomal interaction, loss strong inter-chromosomal interaction, gain strong intra-chromosomal interaction and loss strong intra-chromosomal interaction, detailed information please refer to Additional file 7). The *T*-test was used to evaluate significance of dynamical change of various marks between E2-treated and control conditions (1 Mb resolution), positive and negative T-values are colored by yellow and blue, respectively. In the figure, 0, +1 and −1 represent E2 treated condition vs. control condition at gene body, 5 kb upstream and 5 kb downstream, respectively.

In order to understand the epigenetic influence on four types of interactions and gene regulation, a *t*-test was used to evaluate the significance of changes on six histone marks (H3K4me1, H3K4me3, H3K9me3, H3K27me3, H3K9ac, H3K14ac), Pol-II level and regulatory activity (FAIRE), between the E2-treated and control conditions on genes (~21,136 genes) located in the four types of interacting regions. Two histone marks (H3K4me2 and H3K9me2) and DNA methylation were not included in the analysis due to lack of E2-treated condition. The result (Figure 6) showed that H3K4me3, H3K27me3, H3K9ac and H3K14ac are increased in the E2-treated condition when compared to the control condition for four types of chromosomal changes, while H3K9me3 and Pol-II are decreased upon E2 treatment. Furthermore, the H3K4me1 level remains unchanged between the two conditions. In addition, the tests on the ERα binding site and the chromosomal interaction frequencies show that the former one is highly enriched at the E2-treated condition but the latter one is reduced at the same condition. A similar result was also obtained by performing a Mann–Whitney *U* test on the same data as well as performing a *t*-test on data with 2 Mb window-sizes. It suggests that the results are robust and not subject to change based on different window-size [21] (see Additional file 1: Figure S13,14).

Thus, our results revealed several interesting relationships among chromosomal interaction, histone modifications and gene regulations (Figure 6): 1) H3K4me3, two histone acetylation marks (H3K9ac and H3K14ac), as well as H3K27me3 levels are significantly enriched upon estrogen stimulation. From literature, we knew that the former three histone marks are always enriched in active genes [22] while the latter one is enriched in repressed genes [23]. In particular, both active and repressive marks can coexist led to the “bivalent domain” model and the regions maintain low transcription state [24,25]; 2) H3K4me1 level is not affected by the change of chromosomal interactions (i.e. a strong positive correlation in Additional file 1: Figure S10), H3K4me1 is known for its involvement in enhancer activity especially in the active distal regulatory region; 3) both repressor mark H3K9me3 and Pol-II levels are decreased in the E2-treated condition for all four types of chromosomal interactions. These results suggest that E2-mediated switching change of chromosomal interactions may be epigenetic regulated and influence on both positive and negative gene regulation, as there is increasing H3K4me3 and H3K9ac levels but decreasing H3K9me3 levels after E2-treated in MCF7 cells.

### Characterization of E2/ERα-regulated genes in high frequent and strong differential chromosomal interactions

Next, we wanted to examine the genes in the top 10 regions with the most gain- or loss- interactions upon E2 treatment (Additional file 1: Table S5). There are 79 genes located in these regions and we thus performed GO annotation on those 79 genes (a detailed description in Additional file 9). The results are similar to the top 10 hot regions: GO annotation shows genes are mainly involved in intracellular signaling cascade and in the intracellular receptor-mediated signaling pathway; tissue expression analysis reveals that breast (mammary gland) cancer disease 3^rd^ and mammary gland carcinoma 3^rd^ are significantly enriched, as well as breast cancer is among the most significant disease link to the regions. In particular, a protein interactions study reveals that those genes are potentially co-regulated or co-targeted by a wide range of transcription factors (i.e. more than eighty TFs; Additional file 9) such as FOXO1, FOXO4, FOXJ2, LHX3, HFH3, HFH1, OCT and HNF1. This is also true for genes that are located in the top 10 or 50 hot regions (Table 2; Additional file 2 and Additional file 4), and is consistent with previous observation in Figure 5, the higher the interaction frequency the higher the ERα binding occupancy. Surprisingly, the time-course microarray expression profiles of those 79 genes do not show any major changes across the 12 time points (0 hour to 32 hours; Additional file 1: Figure S15), except for a few genes after 20 hours of E2 treatment.

To further test whether large scale chromosomal reorganization affects gene expression, we compared the number of differential expressed genes in these differential interaction regions to the overall number of differential expressed genes after E2 treatment in MCF7 cells. A Chi square analysis showed that regions gained or lost intra-chromosomal interactions, however inter-chromosomal interactions were no more subject to expression change than overall genes. The p values of the Chi square tests were as follows: loss intra-chromosomal interaction vs. overall -- p < 0.0001; gain intra-chromosomal interaction vs. overall -- p < 0.0001; loss inter-chromosomal interaction vs. overall -- p = 0.113; and gain inter-chromosomal interaction vs. overall -- p = 0.619. This result suggests distinct roles of inter-chromosomal interactions and intra-chromosomal interactions in gene regulation. A small portion of the gene expression was affected by chromosomal interaction changes indicating that chromatin organization is one of the many factors involved in gene regulation.

## Discussion

Although recent advances in high throughput technologies (i.e. ChIP-seq and DNase-seq) have been effectively applied to map transcription factors binding sites and histone modifications on the linear DNA sequence, in the one-dimensional (1D) regulatory view, these techniques are not sufficient to provide information of how gene expression is transcriptionally modulated in the three-dimensional (3D) chromatin architecture. Two recent pioneering studies [3,16] developed a new Hi-C protocol and revealed the extensive genomic interactions. However, they are limited on a single time point Hi-C experiment which can only depict a static chromatin structure. By applying this new emerging technology on a breast cancer model system, before and after estrogen treatment on MCF7 (an ERα positive breast cancer cell line), we comprehensively analyzed the Hi-C data performed in our laboratory and revealed a dynamic chromatin re-organization upon E2 stimulation resulting in altered gene regulation and their associations with epigenetic modification states. Our work provides further insight into the effect of chromatin interaction on E2/ERα regulated downstream genes in breast cancer cells.

Several studies support a notion that long-range regulation of many critical genes may be involved in both normal development and disease [5,10,11,26-28]. A recent study showed that genes with similar functions form a multi-gene complex through chromatin interactions and cooperatively expressed at a high level [29]. Interestingly, by GO analysis, we found that breast cancer associated genes are enriched in highly interactive regions (*i.e.* chromosomes 17 and 20) in MCF7 cells. Our results indicate that the disease related genes might be co-upregulated in breast cancer cells due to the high order structure changes of the chromatin (Table 2). Genes in low chromosomal interaction frequency regions may not contribute to the development of cancer, but genes in high interaction frequency regions (> = 30%) may be essential to human disease such as breast cancer.

Our study also suggests that the dynamic chromosomal interaction switches are frequently occurring in the chromosomal interaction hot regions (i.e. chromosomes 17 and 20; Table 2) after E2 treatment. Those regions contain genes not only highly associated with breast cancer but also actively participating in signaling cascade and signaling pathways. Though gene expression levels are not significantly changed after E2 treatment at the interaction hot regions, they interact with a wide range of TFs such as FOXO1. Thus, chromosome interaction hot regions are potentially very important to functional genome regulation because they bear active chromosome interaction upon E2 stimulation, and the regions may function as a harbor to recur many TFs for initiating more activities in the downstream gene regulations such as histone modifications.

## Conclusions

Our study is among one of the first attempts to profile genome-wide dynamic chromatin re-organization in response to environmental changes. We observed both extensive looping and de-looping events in MCF7 cells after E2 treatment, and discovered that dynamic changes of both inter- and intra-chromosomal interactions do exist with predominate inter-chromosomal changes. Further, we found both active (H3K4me3, H3K9ac) and repressive (H3K27me3) histone modification changes associated with interaction gain or loss. This result indicated a dual-role of chromatin structure change on transcriptional regulation, one part of the chromatin territories forms a co-activate complex while the other part of the chromatin forms a co-repressive complex. We linked the dynamic organization to major transcription factors associated with disease development, in this case estrogen receptor α, and transcriptional regulation. In addition, a strong negative correlation between interaction and nucleosome density (FAIRE level) provided evidence of the cooperative regulation of genes. These results strongly suggest that environmental stimulation can trigger the re-organization of the chromatin, which can further impact regulation of critical genes. The elucidation of the association between chromatin interaction and critical genes may shed light on the underlying mechanisms of disease development and progression.

## Methods

### Hi-C Method

MCF-7 cells were grown in a medium with 5% charcoal/dextran-stripped fetal bovine serum (HyClone) for 24 hours and then stimulated with 17β-estradiol (E2, 70 nM) or DMSO (Control) for 1 hour. Treated cells were then subjected to Hi-C as previously described [3]. Briefly, cells were cross-linked with 1% formaldehyde. DNA was digested with HindIII to create a 5’ overhang. The 5’ overhang was filled within including a cytosine-biotinylated residue. The resulting blunt-end fragments were ligated by T4 ligase under diluted conditions that favor ligation events between the cross-linked DNA fragments. Ligated DNA was then de-cross-linked (overnight at 65°C) and purified by phenol extraction procedures. Ligated DNA (5 μg) was randomly sheared by a nebulizer supplied with the Illumina paired-end sample preparation kit, then applied to paired-end sequencing as per the manufacturer’s instructions by using the Illumina Genome Analyzer IIA. For each time point, four technical replicates of Hi-C experiments were performed. Correlation analysis and scatter plot of these replicates are shown in Additional file 1: Figures S16 and Table S6. In this study, all reads of replicates were combined for downstream data analysis, a total of ~125 and ~111 million raw paired 51 bp sequence reads were obtained for control and E2 treatment respectively. After aligning to the Human Genome Assembly (NCBI build 36.1/hg18) using the ELAND algorithm and removing redundant reads in order to reduce DNA amplification and system biases, a total of ~92 and ~80 million reads were uniquely mapped to the human genome. In order to minimize the effect by genomic regions amplified in MCF7, all duplicated pair-end interactions were removed.

### Chromosome interaction frequency

After pre-processing [12], normalization and transformation of Hi-C interaction data to Z-scores, distribution of chromosomal interaction frequencies of both control (T0) and E2 treated experiments (T1) were studied in 1 Mb resolution. Specifically, for every 1 Mb window-size of chromosome regions, the number of interactions (Z-scores not equal to zero) between the region and the rest of the chromosome regions were counted. The chromosomal interaction frequency of the region was then calculated as the counted number of interactions in the region divided by the total number of chromosome regions (e.g. with 1 Mb resolution, there are 3,029 chromosome regions in the human genome). Z-score of intra- or inter-chromosomal interaction matrices (raw interaction counts in 1 Mb resolution divided by the average expected level of interactions [12]) are illustrated in both the genome-wide heat map and chromosome specific intra-chromosomal interaction heat map. Relative ratios [30], *i.e.* the difference of exponential Z-score between the two conditions divided by the average of Z-scores of chromosome interaction changes between control and E2 treated conditions are used to investigate the dynamic feature of chromosomal interactions between control and E2 treated experiments, where zero elements (no interaction) remain as zero.

### Processing ChIP-seq of histone modifications, ERα and Pol-II, MBD-seq and FAIRE-seq data

Histone modification datasets (e.g., H3K14ac, H3K27me3, H3K4me1, H3K4me2, H3K4me3, H3K9ac, H3K9me2 and H3K9me3), DNA methylation, DNA accessible regions (FAIRE), Pol-II and ERα ChIP-seq data under both E2 treated and control conditions were publically collected [13,20,31]. Preprocessing of the above-mentioned ChIP-seq datasets were accomplished by BALM [32] such as read counts and peak identification. Enrichment test (*t*-test and Mann–Whitney *U* test) between E2 treated and control conditions was adopted from a previous study [33]. Gene expression profiles for E2 stimulated MCF-7 cells were taken from the study [17] and preprocessed by MArray [34]. The DAVID tool was used to perform functional annotation of gene analysis [18].

### Validations of ERα regulated interaction by quantitative 3C-PCR

Chromosome conformation capture assay combined with quantitative PCR analysis (3C-qPCR) was performed as previously described [19]. Briefly, MCF-7 cells were treated with E2 (70 nM) for 1 hr and then fixed with 1% formaldehyde. Chromatin was digested using BbvCI (for ESR1) and BamHI (for target loci), and then ligated by T4 DNA. Ligated DNA was then de cross-linked (overnight at 65°C) and purified by classical phenol extraction procedures. Real-time PCR was performed on a 7500 Real-Time PCR System apparatus (Applied Biosystems) using the TaqMan technology (QuantiTect Probe PCR Master Mix, Qiagen). We used a 59FAM-39BHQ1 oligonucleotidic probe (IDT). Five loci, including THRAP1 (17q23), INTs2 (17q23), C16orf65 (16p12), CADPS (3p14) and ZIM2 (19q13), were chosen to examine the interactions. We utilized ERα binding sites (ERαBS) located at the 20q13 region as the bait to interrogate the interactions between ERαBS and promoter regions of three loci. To rule out the possibility of false-negative looping occurrence caused by unsuccessful 3C assay, we pooled three human bacterial artificial clones (BAC), mapping the interested regions as the positive control to the 3C assay. BACs were also used to examine the primer efficiency. Ct values obtained for each chimerical ligation fragment were processed using parameters of a standard curve (slope and intercept) from BAC to obtain quantification values that were normalized to a GAPDH loading control. Results are presented as the mean of triplicates with standard derivation.

## Competing interests

The authors declare they have no competing interests.

## Authors’ contributions

JW conceived and designed the study, performed data analysis, interpreted results and drafted manuscript; VXJ conceived, designed and coordinated the study, wrote the manuscript; XL participated in data analysis, results interpretation and wrote the manuscript; PH and HH performed the validation. KH, JP, TH helped draft the manuscript. All authors read and approved the final manuscript.

## Authors’ information

Junbai Wang and Xun Lan Co-1st authors.

## Supplementary Material

Additional file 1 **Additional Figures and Tables. **Contains all additional tables and figures. Click here for file

Additional file 2 **DAVID results for genes in chromosomal interaction hot regions. **Contains DAVID results of 69 genes located in the top 10 chromosomal interaction hot regions. Click here for file

Additional file 3 **DAVID results for genes in chromosomal interaction cold regions. ** Contains DAVID results of 45 genes located in the top 10 chromosomal interaction cold regions. Click here for file

Additional file 4 **DAVID results for genes in chromosomal interaction hot regions. **Contains DAVID results of 280 genes located in the top 50 chromosomal interaction hot regions. Click here for file

Additional file 5 **DAVID results for genes in chromosomal interaction cold regions. **Contains DAVID results of 500 genes located in the top 50 chromosomal interaction cold regions. Click here for file

Additional file 6 **Comparing 69 genes at interaction hot regions with Fullwood’s results.** Contains a comparison between 69 genes at the top 10 chromosomal interaction hot regions and Fullwood’s results. Click here for file

Additional file 7 **Strong chromosomal interaction changes. **Contains chromosome regions with strong interaction changes under the E2 treated condition (e.g. Zscore > 1 and Relative ratios = 2). Click here for file

Additional file 8 **Additional Figure. **Contains an additional figure that shows the correlation between histone modification and chromosomal interaction frequency for all interaction regions in 1Mbp resolution. The order of chromosome regions is sorted by their interaction frequency at the control condition. Click here for file

Additional file 9 **Genes of top 10 regions with the most gained and lost interactions according to four types of strong chromosomal interaction changes. **Contains results of functional annotation of genes in the top 10 most frequent dynamical chromosomal interaction regions. The regions are estimated from four types of strong chromosomal interaction changes. Click here for file
