# Supplementary material for: Genome-wide analysis uncovers high frequency, strong differential chromosomal interactions and their associated epigenetic patterns in E2-mediated gene regulation
Source: BMC Genomics. 2013 Jan 31;14:70. doi: 10.1186/1471-2164-14-70 (PMC3599885; doi:10.1186/1471-2164-14-70)
Supplement: Additional file 1 — Additional Figures and Tables. Contains all additional tables and figures. [file 1471-2164-14-70-S1.docx]

**Supplementary to “Genome-wide analysis uncovers high frequency, strong differential chromosomal interactions and their associated epigenetic patterns in E2-mediated gene regulation”**

Junbai Wang, Xun Lan, Pei-Yin Hsu, Hang-Kai Hsu, Kun Huang, Jeffrey Parvin, Tim H.-M. Huang and Victor X. Jin

**Supplementary Results**

***Correlation between E2-mediated chromosomal interaction frequency and epigenetic modifications***

To determine correlations among chromosomal interaction frequency, epigenetic marks and transcriptional regulation, eight publicly available histone marks (H3K4me1, H3K4me2, HK4me3, H3K9me2, H3K9me3, H3K27me3, H3K9ac, H3K14ac), DNA methylation, Pol-II level and regulatory activity (FAIRE) were used to calculate the log transformation of read counts for genes within every 1 Mb window-size region. Here ~16% of 1Mb chromosome regions (480) were excluded from the analysis because there is no gene in these regions. In order to determine the role of each specific regulatory region for any given gene, we further divided each gene to three regulatory regions in reference to 5’ transcription start site (5TSS), 5 Kb upstream, 5 Kb downstream and gene body. Then the mean of log transformed read counts for each part at the 1Mb chromosome region were computed and displayed in a heat map (**Additional file 7**), in which the order of chromosome regions was sorted by the interaction frequency at the control condition. The result showed that there is a clear separation between the interaction hot regions (regions with the highest chromosomal interaction frequency; lowest panel of **Additional file 7**) and the interaction cold regions (regions with the lowest chromosomal interaction frequency; top panel of **Additional file 7**).

By a more close examination of the top 10 hot regions (**Figure S9**), all histone modifications (i.e., H3K14ac, H3K9ac, H3K9me2, HeK9me3, H3K27me3, H3K4me1, H3K4me2 and H3K4me3) and Pol-II level are highly enriched in all of three regulatory regions regardless of the experimental conditions. This is also true for the DNA methylation and FAIRE levels (an evidence of easier accessible regulatory regions). In the contrary, for the top 10 cold regions, all histone modifications, DNA methylation and Pol-II levels are very weak as well as the FAIRE levels (an evidence of harder accessible regulatory regions). Additionally, we did not find any ERα binding in the 10 interaction cold regions for both E2-treated and control conditions, while at least six ERα binding sites were found in every of top 10 interaction hot regions. These results demonstrated that chromosome regions with the intermediate interaction frequency may bear regional-specific histone modification and Pol-II level. However, for chromosome regions with extremely low or high interaction frequency, they share extremely low or high histone modification (Pol-II levels), respectively. Thus, chromosomal interaction frequency may play a functional role in gene regulation due to its close association with epigenetic modifications and Pol-II levels.

Our fine-scale examination of a heat map of correlation coefficient matrices built upon on genome-wide integrated ‘omics data (**Figure S10**), it revealed several interesting relationships: 1) there is a strong association between the interaction frequency and the accessibility of regulatory regions such as the higher interaction frequency the easier accessible regulatory region (FAIRE levels); 2) a strong positive correlation between interaction frequency and H3K9me2 (a repressive histone mark) level at the control conditions; 3) the number of ERα binding sites under the E2-treated condition is positively correlated with H3K4me1 level (an enhancer histone mark) at the control condition; 4) there is no significant change of histone modification level and regulatory region accessibility between the E2-treated and control conditions, except for H3K4me3 and H3K9ac levels which are often enriched in active promoters. Those results suggested that both chromosomal interaction frequency and E2-mediated gene regulation are associated with histone modification states as well as with the accessibility of regulatory regions.

**Supplementary Figures**

**Figure S1a**. **A genome-wide chromosomal interactions matrix of 1 Mb resolution in E2-treated condition.**

Z-score of intra- and inter-chromosomal interaction matrices (i.e. raw Hi-C interaction counts in 1Mb resolution divided by the average expected level of interactions) are displayed in a genome-wide heat map, in which positive and negative Z-scores are colored by red and green color that indicate the observed chromosome region has higher and lower interaction frequency than the average, respectively.


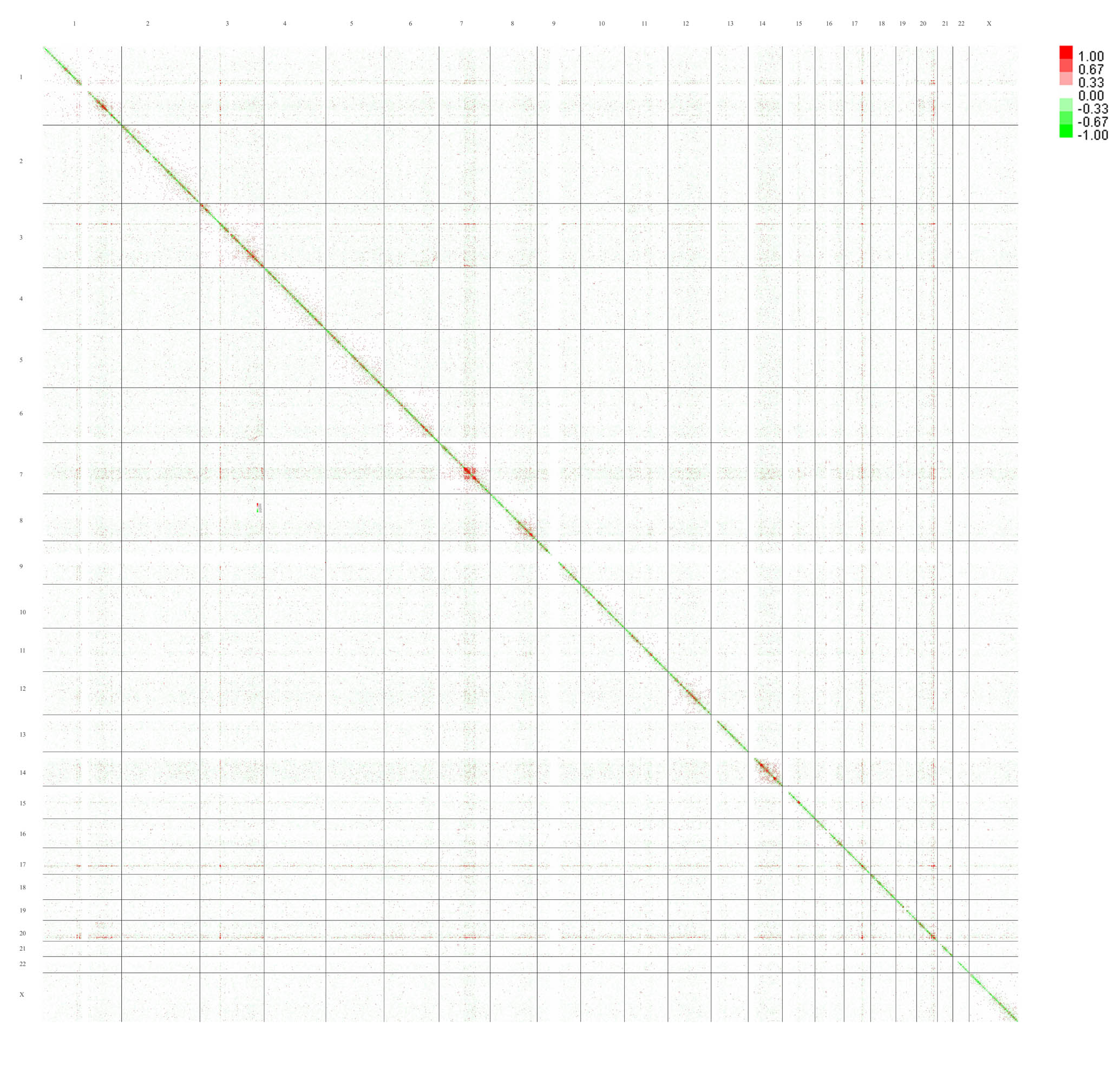


**Figure S1b**. **A genome-wide chromosomal interactions matrix of 1 Mb resolution in control condition.**

Z-score of intra- and inter-chromosomal interaction matrices (i.e. raw Hi-C interaction counts in 1Mb resolution divided by the average expected level of interactions) are displayed in a genome-wide heat map, in which positive and negative Z-scores are colored by red and green color that indicate the observed chromosome region has higher and lower interaction frequency than the average, respectively.


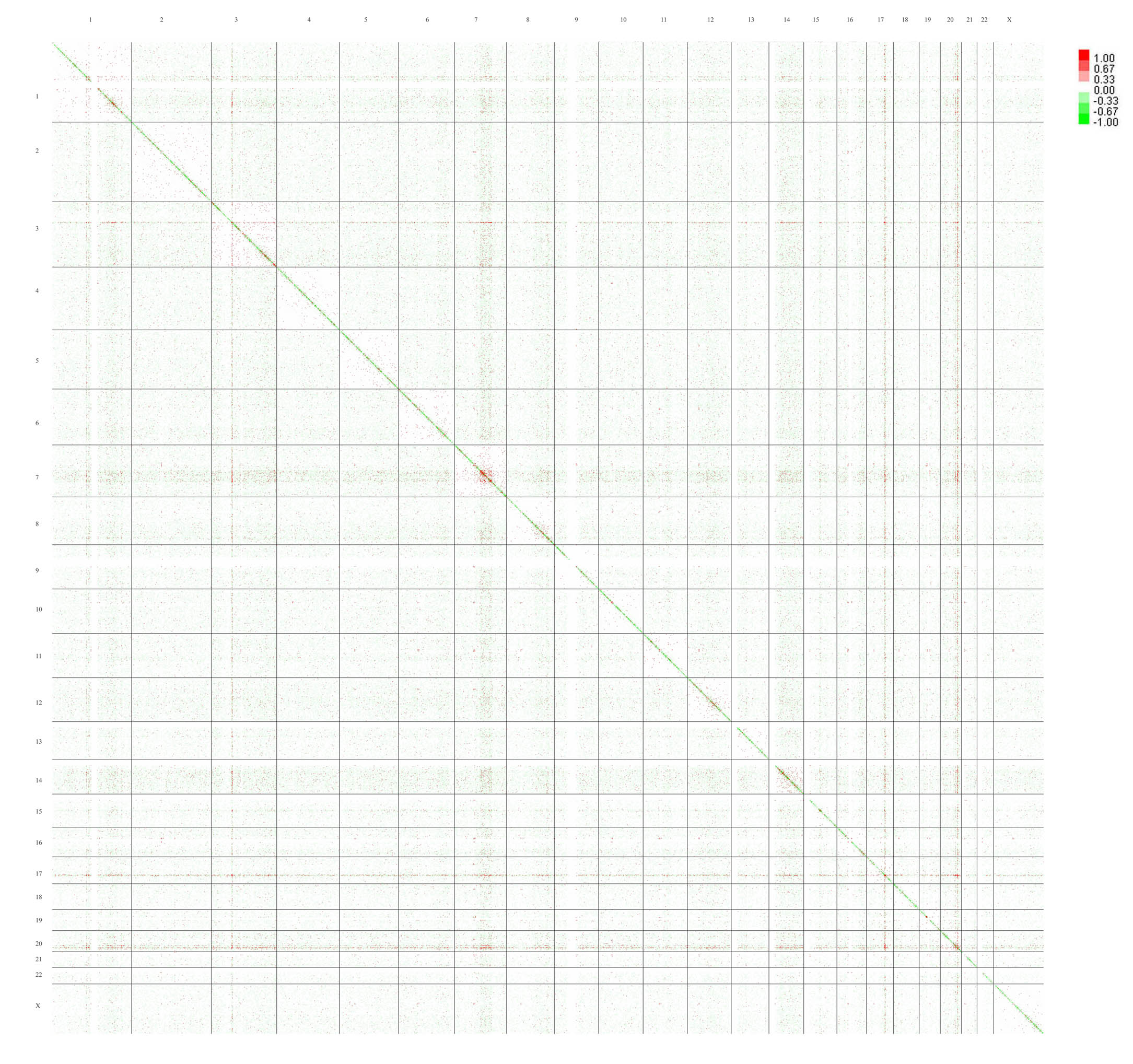


**Figure S2**. **Chromosomal interaction hot regions in 1 Mb resolution.**

Upper panel: intra-chromosomal interaction for chromosome 3 at control condition; down panel: intra-chromosomal interaction for chromosome 3 at E2-treated condition; right panel, red smooth line represents detected number of ERα binding sites in the region, and blue smooth line is the maximum read counts in the region; left panel, positive and negative Z-scores are colored by red and green color, which indicate the observed chromosome region has higher and lower interaction frequency than the average, respectively.


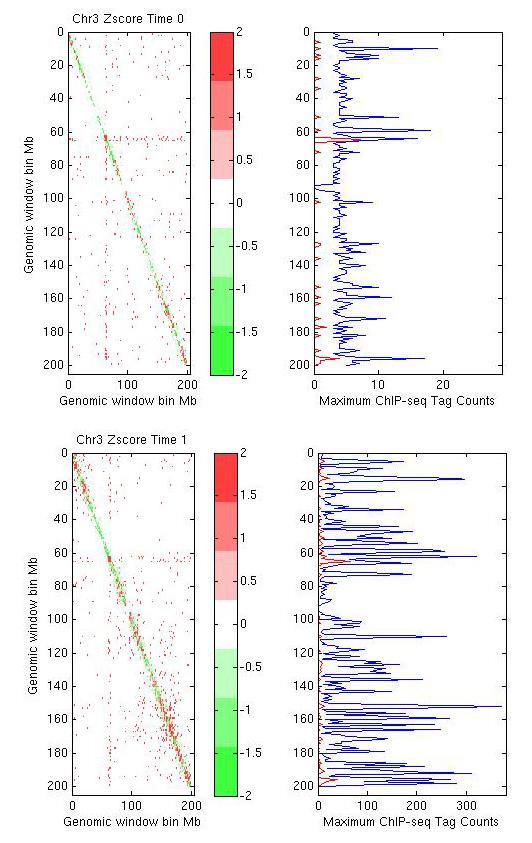


**Figure S3**. **Chromosomal interaction hot regions in 1 Mb resolution.**

Upper panel: intra-chromosomal interaction for chromosome 17 at control condition; down panel: intra-chromosomal interaction for chromosome 17 at E2-treated condition; right panel, red smooth line represents detected number of ERα binding sites in the region, and blue smooth line is the maximum read counts in the region; left panel, positive and negative Z-scores are colored by red and green color, which indicate the observed chromosome region has higher and lower interaction frequency than the average, respectively.


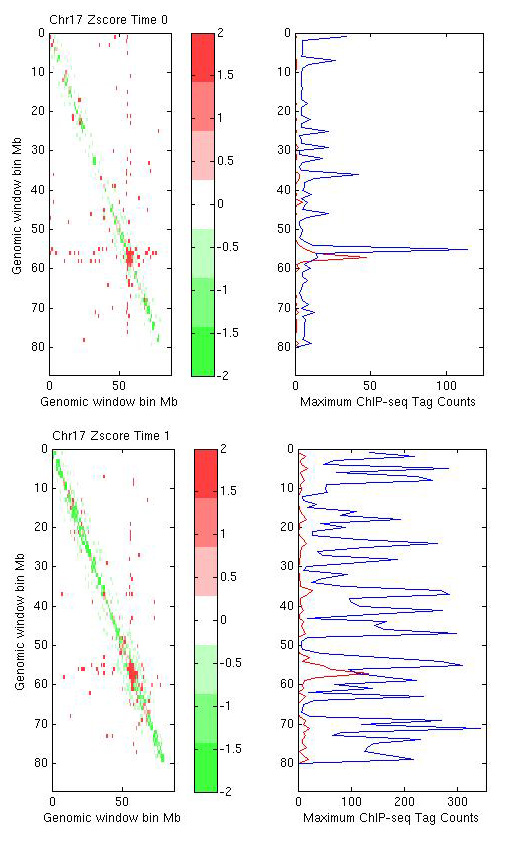


**Figure S4**. **Chromosomal interaction hot regions in 2 Mb resolution.**

Upper panel: intra-chromosomal interaction for chromosome 3 at control condition; down panel: intra-chromosomal interaction for chromosome 3 at E2-treated condition; right panel, red smooth line represents detected number of ERα binding sites in the region, and blue smooth line is the maximum read counts in the region; left panel, positive and negative Z-scores are colored by red and green color, which indicate the observed chromosome region has higher and lower interaction frequency than the average, respectively.

**
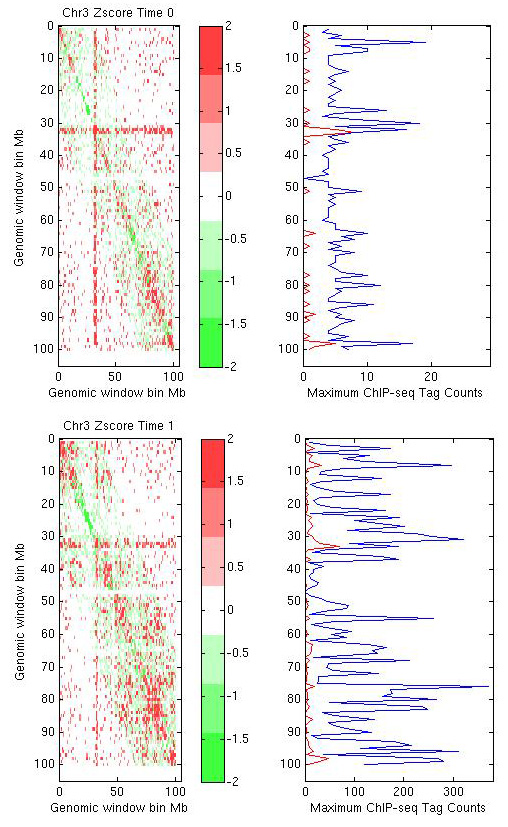
**

**Figure S5**. **Chromosomal interaction hot regions in 2 Mb resolution.**

Upper panel: intra-chromosomal interaction for chromosome 17 at control condition; down panel: intra-chromosomal interaction for chromosome 17 at E2-treated condition; right panel, red smooth line represents detected number of ERα binding sites in the region, and blue smooth line is the maximum read counts in the region; left panel, positive and negative Z-scores are colored by red and green color, which indicate the observed chromosome region has higher and lower interaction frequency than the average, respectively.


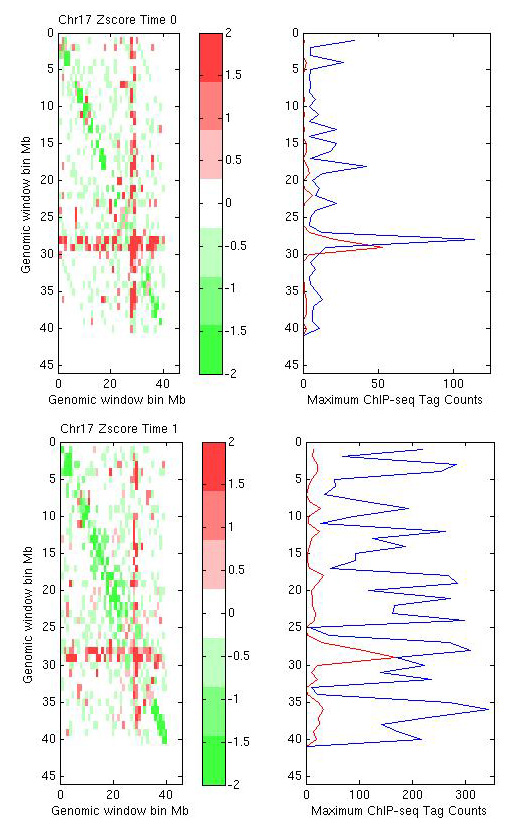


**Figure S6**. **Chromosomal interaction hot regions in 2 Mb resolution.**

Upper panel: intra-chromosomal interaction for chromosome 20 at control condition; down panel: intra-chromosomal interaction for chromosome 20 at E2-treated condition; right panel, red smooth line represents detected number of ERα binding sites in the region, and blue smooth line is the maximum read counts in the region; left panel, positive and negative Z-scores are colored by red and green color, which indicate the observed chromosome region has higher and lower interaction frequency than the average, respectively.


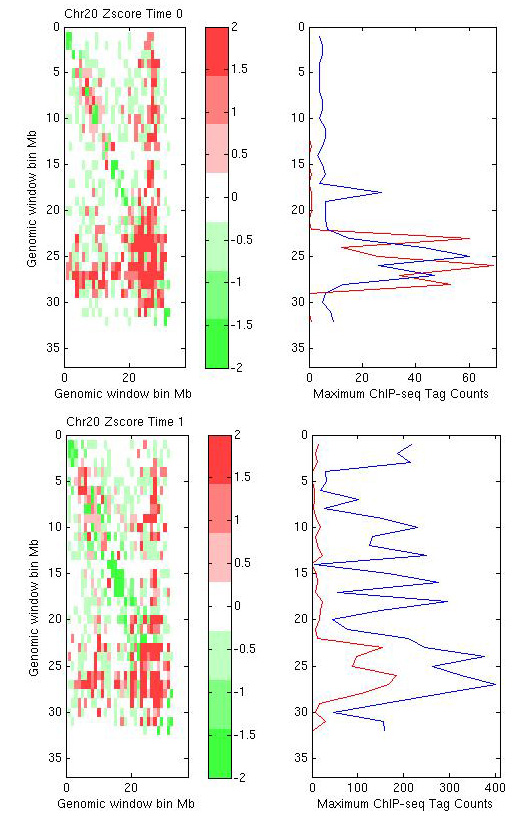


**Figure S7. Validations of Hi-C data by quantitative 3C-PCR (3C-qPCR).**

MCF-7 cells were treated with E2 (70 nM) for 1 hr and then subjected to quantitative 3C-PCR. Five loci, including *C16orf65* (16p12), *INTS2* (17q23), *CADPS* (3p14), *THRAP1* (17q23), and *ZIM2* (19q13), were chosen to examine the promoter-enhancer interactions. We utilized ERa binding sites (ERaBS) located at 20q13 region as the bait to interrogate the interactions between ERaBS and promoter regions of five loci. The *de novo* looping formations were observed in C16orf65, INTS2, CADPS and THRAP1 loci upon 1 hr E2 treatment. Each validated loci were done in two biological replicates and three technical replicates per biological replicate. The Y-axis label means how often the selected loci interacted with the rest of chromosomes.

**Figure S8**. **A heat map of time-series gene expression profiles in top 10 hot interaction regions (1 Mb resolution).**

Here Z-scores of time-series expression levels (after E2-treatment) of 69 genes that located in the top 10 hot interaction regions are shown in color coded heat map. In the figure, red and blue colors represent positive (up regulation) and negative (down regulation) Z-scores, respectively.


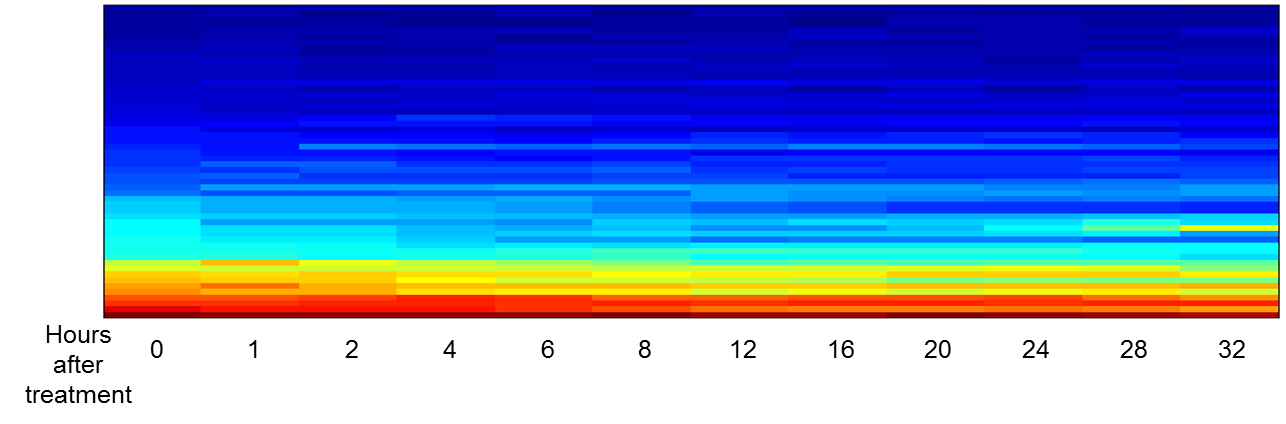


**Figure S9**. **Correlation between histone modification and chromosomal interaction frequency (1Mb resolution) for top 10 hot and cold regions.**

Here all data are log transformed then visualized by heat map. The order of matrices is sorted by chromosomal interaction frequency at control condition. The lower panel is the top 10 hot regions and the upper panel is the top 10 cold regions.


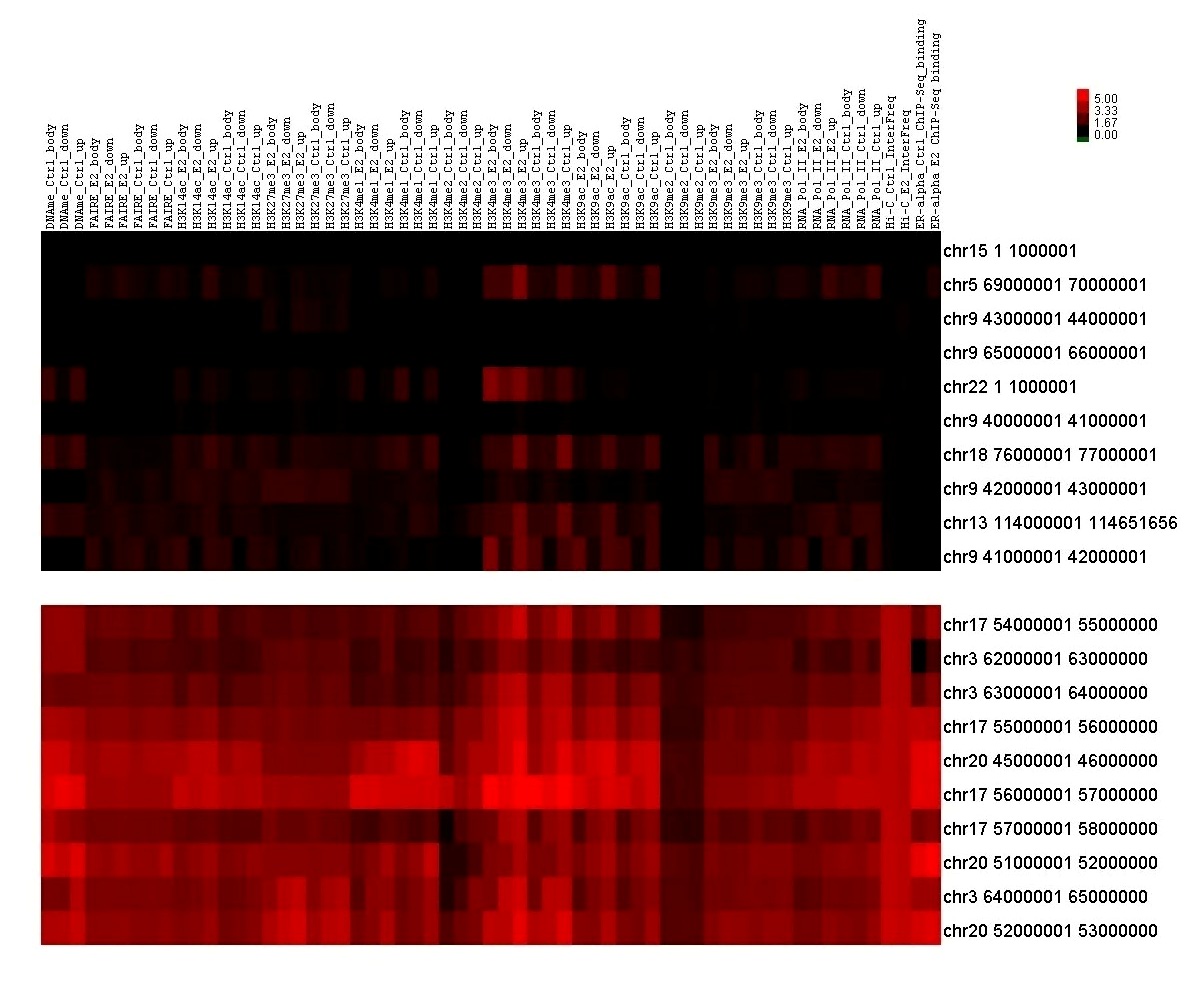


**Figure S10**. **Correlation coefficient matrices for epigenetic markers and chromosomal interaction frequency (1Mb resolution).**

Here the log transformed mean read counts of 5Kb upstream, 5Kb downstream and gene body for every epigenetic marker is used. Then, the correlation coefficients between epigenetic marks and interaction frequency are computed. The results are illustrated in a heat map where light color represents high correlation and dark color means low correlation. In the figure, C0, C+1 and C-1 (E0, E+1 and E-1) represent control (E2-treated) condition at gene body, 5kb upstream and 5kb downstream, respectively; CI and EI means interaction frequency under control and E2-treated condition, respectively; CM and EM represent ER-alpha binding motifs at control and E2-treated condition.


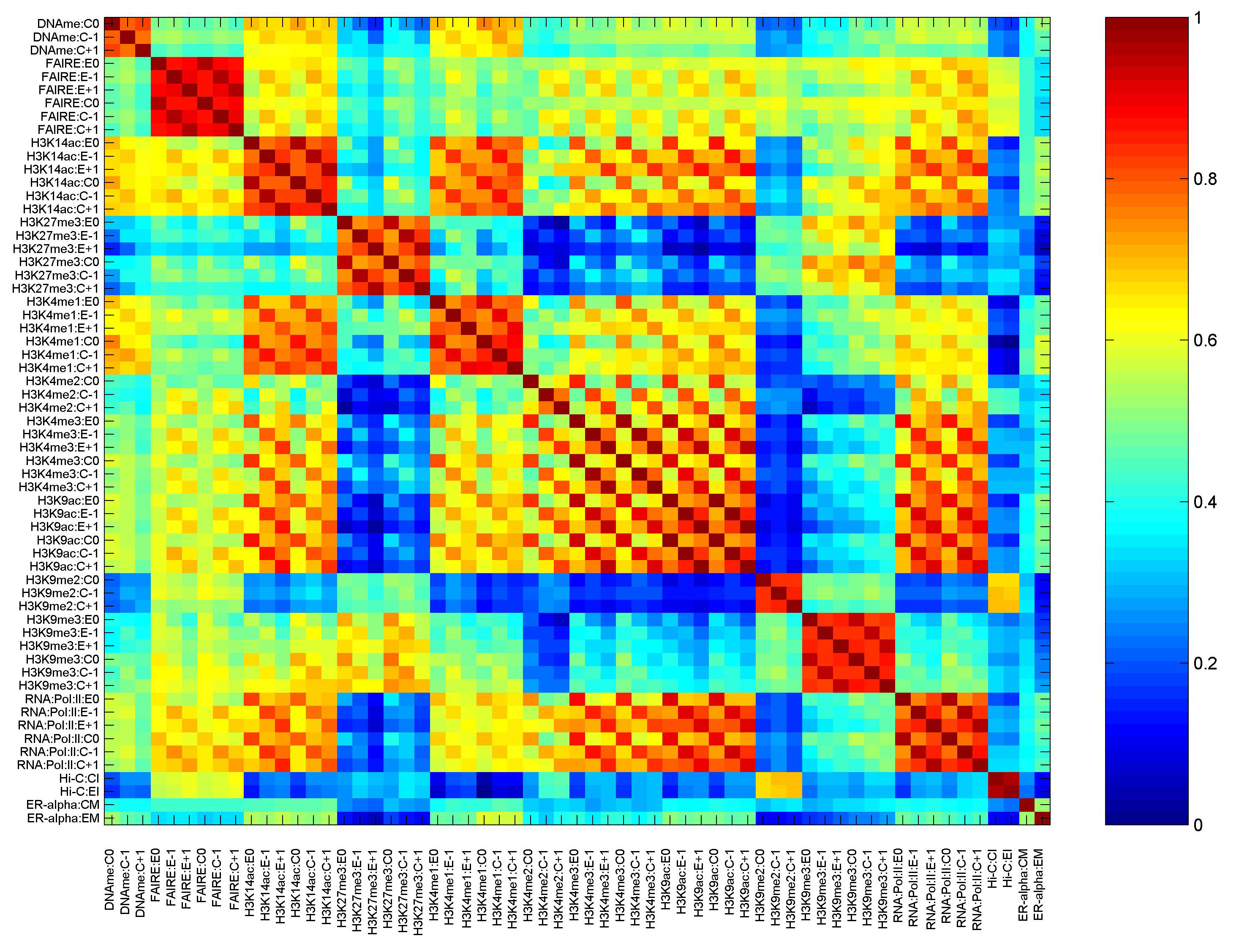


**Figure S11**. **Distribution of relative ratios of chromosome interaction changes (2Mb resolution).** Upper panel: Histogram of relative ratios (chromosomal interaction changes, E2-treated *vs* control condition). Lower panel: sorted relative ratios, red smooth line is relative ratio equals 0.67 (e.g., a 2 fold change) and green smooth line is relative ratio equals 1.33 (e.g., a 5 fold change). Non-interaction elements are excluded from analysis such as Z-score equals 0 in both control and E2-treated interaction matrices. A 10-fold interaction change is expected when the relative ratio equals 1.63, gain and lost interactions are equivalent to the relative ratio 2 and -2, respectively.

**
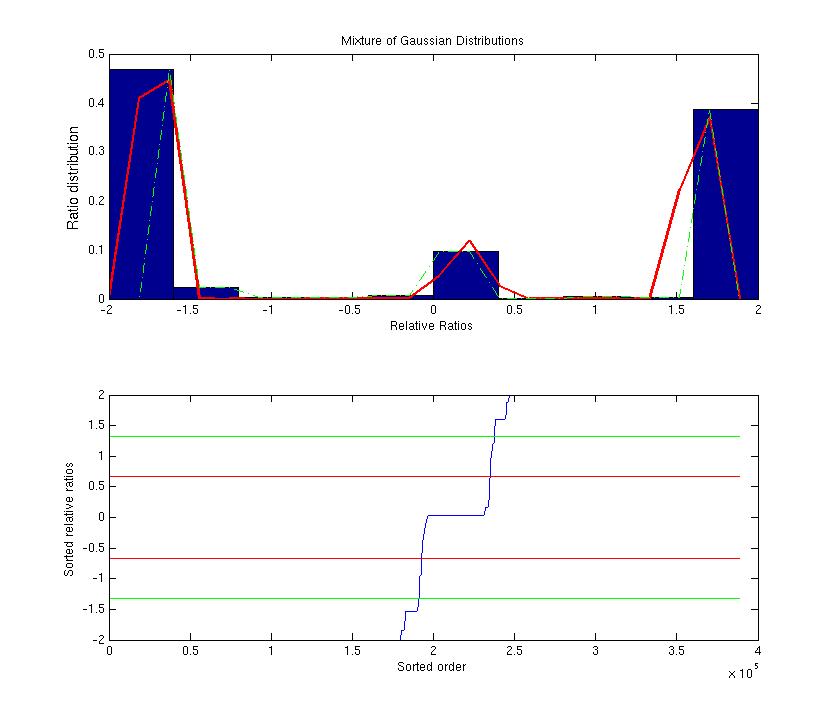
**

**Figure S12**. **Dynamical changes of chromosomal interactions between control and E2-treated conditions (2 Mb resolution).**

Number of gained (i.e. red smooth line, positive value) and lost (i.e. blue smooth line, negative value) interactions between control and E2-treatd conditions are calculated for every 2Mb region of human genome based on the four types of the strongest chromosomal interactions (i.e. strong differential gain or loss chromosomal intra or inter interactions).

**
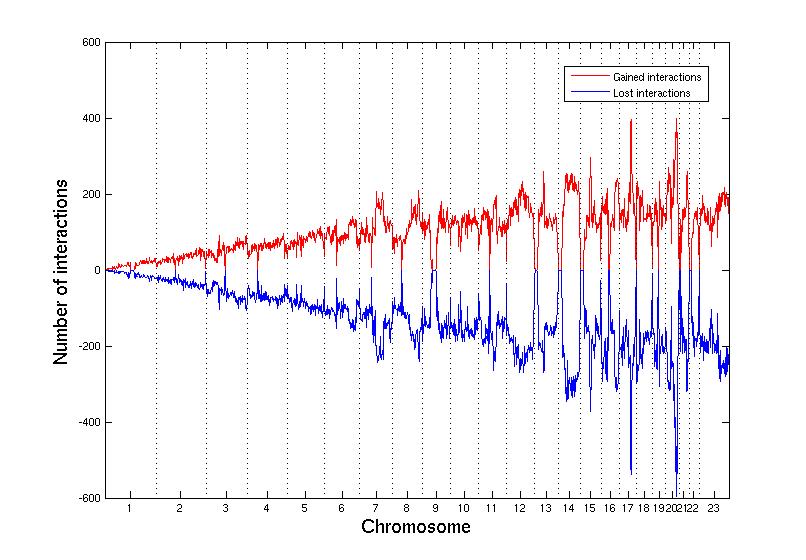
**

**Figure S13**. **A heat map of dynamic change of histone modifications between control and E2 treated experiments for four types of chromosomal interactions (1Mb resolution)**.

Here Z-values are obtained by perform Mann-Whitney U test for genes that were chosen by the four types of strong chromosomal interactions in section 3 (e.g., gain strong new inter-chromosomal interaction, loss strong inter-chromosomal interaction, gain strong new intra-chromosomal interaction and loss strong intra-chromosomal interaction, detailed information please refer to Additional file 6). Mann-Whitney U test was used to evaluate significance of dynamical change of various biomarkers between control experiment and E2 treated experiment. Yellow color and blue color represent positive and negative Z-values, respectively. In the figure, 0, +1 and -1 represent E2 treated condition vs. control condition at gene body, 5kb upstream and 5kb downstream, respectively.


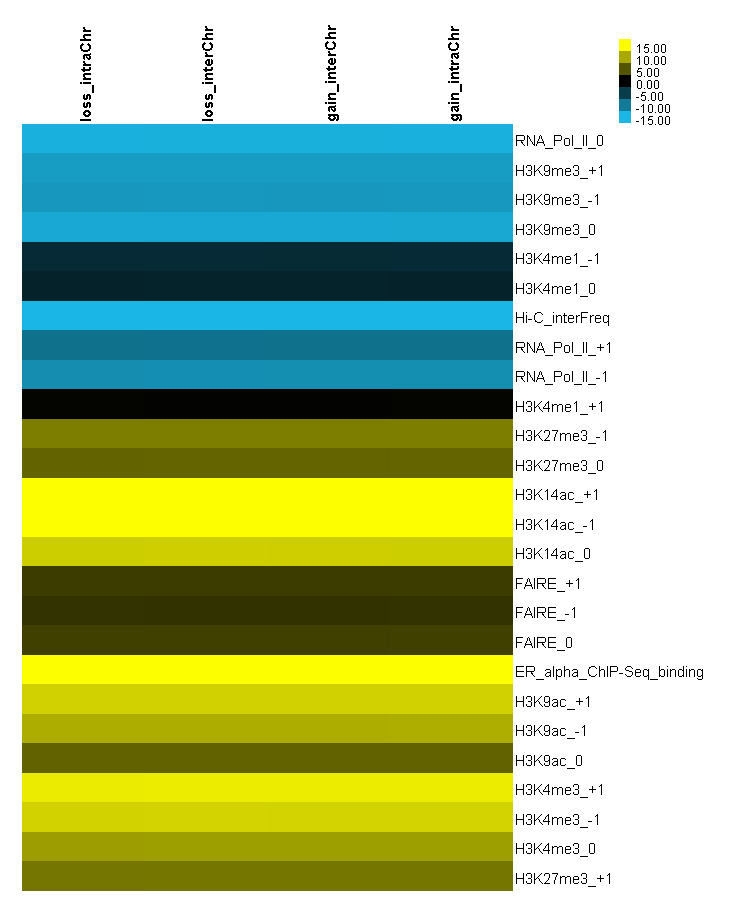


**Figure S14**. **A heat map of change of histone modifications between E2-treated and control conditions for four types of chromosomal interactions (2Mb resolution).**

Here T-values are obtained by perform t-test for genes that were chosen by the identified four types of chromosomal interaction changes (e.g., gain strong inter-chromosomal interaction, loss strong inter-chromosomal interaction, gain strong intra-chromosomal interaction and loss strong intra-chromosomal interaction. The T-test was used to evaluate significance of dynamical change of various marks between E2-treated and control conditions, positive and negative T-values are colored by yellow and blue, respectively. In the figure, 0, +1 and -1 represent E2 treated condition vs. control condition at gene body, 5kb upstream and 5kb downstream, respectively.


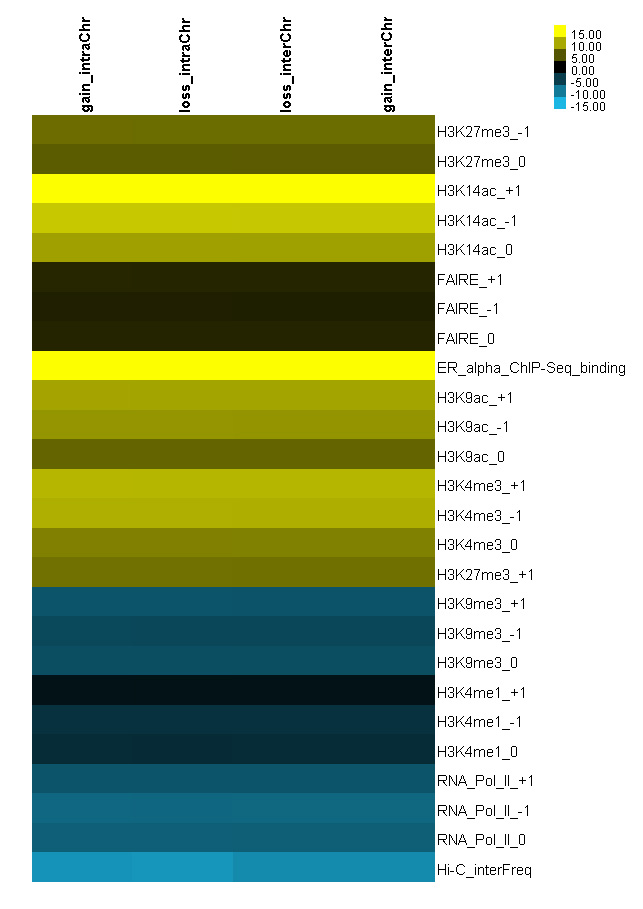


**Figure S15**. **Time-course gene expression profiles after E2 treatment for genes included in the top 10 most frequent interaction changes after E2 treatment (Table S6; 1Mb resolution).**

Here gene expression levels were log transformed and normalized to Z-scores (have variance one and mean equal zero). Red and blue colors represent positive (up regulation) and negative (down regulation) Z-scores, respectively.


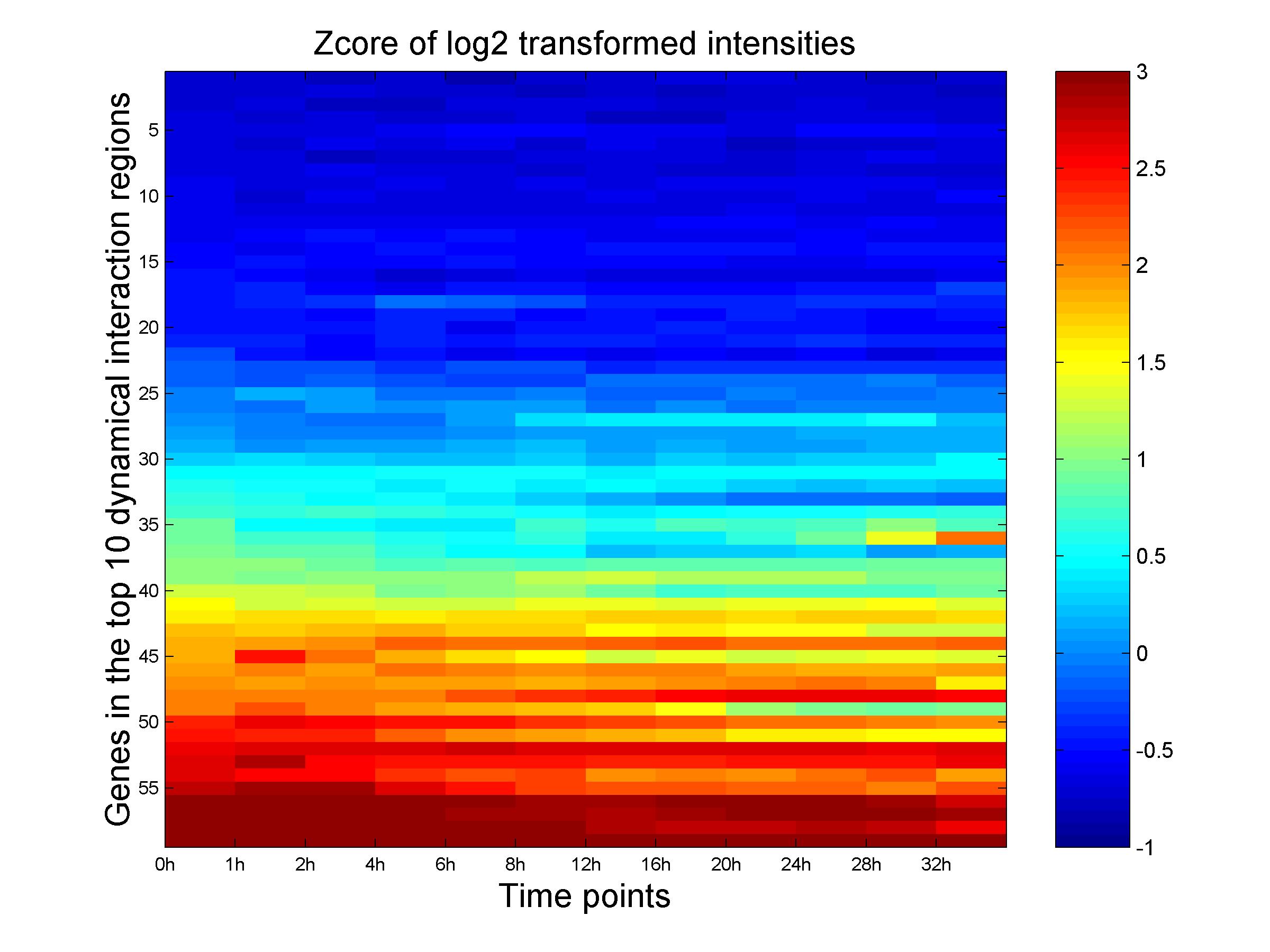


**Figure S16**. **Correlation between replicates of Hi-C experiments.**

Two example scatter plots of log read counts of 1000bp bins in different replicates (for detailed correlation of each pair of replicates see Table S7).


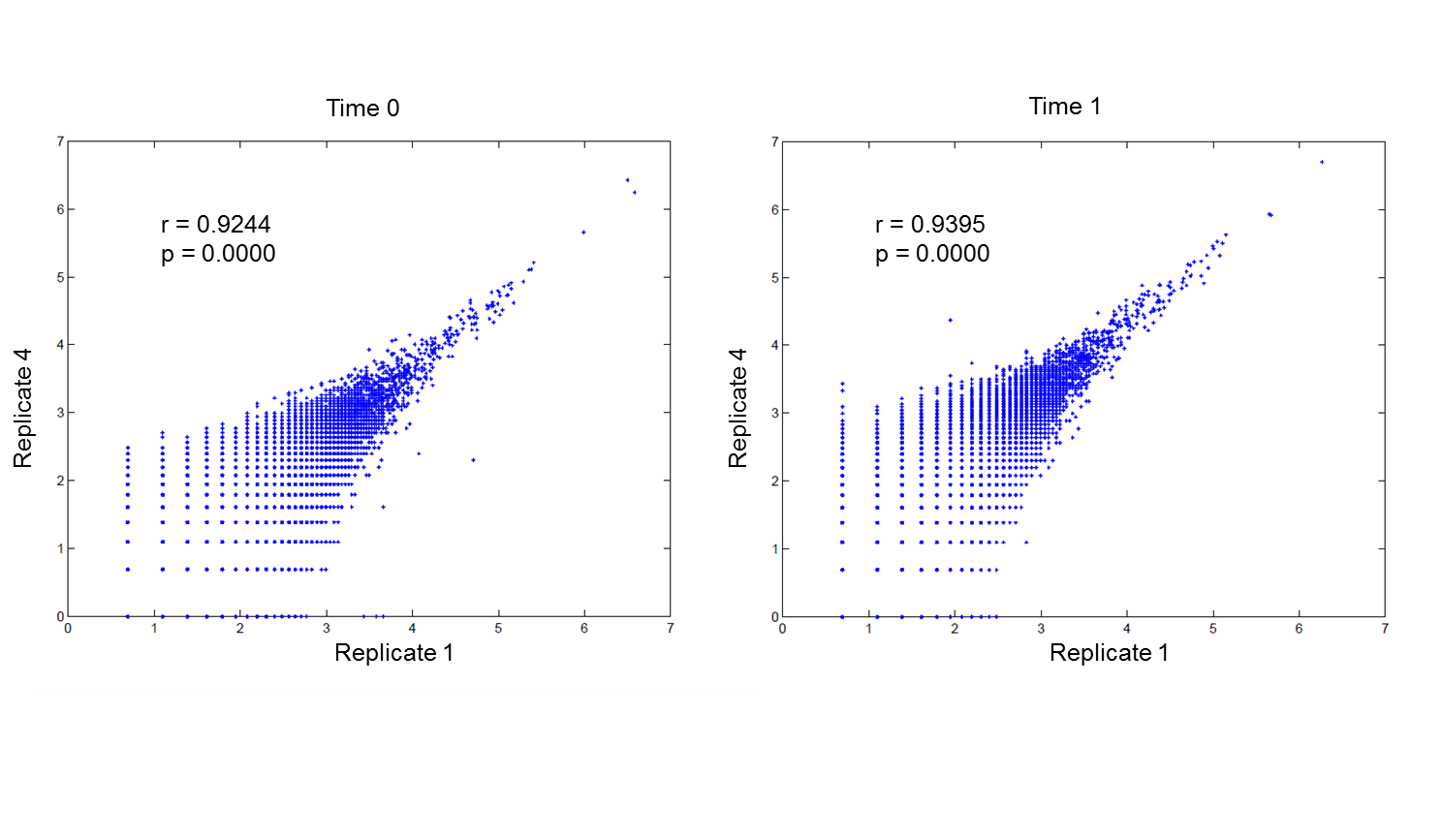


**Supplementary Tables**

**Table S1**. Distribution of chromosomal interaction frequency in the human genome (2Mb resolution), where the number of regions with interaction frequency greater than and equal to 1%, 5%, 10%, 20%, 30%, 40%, 50% and 60% are shown respectively.

| **Chromosomal Interaction frequency ( >=)** | **Number of regions**  **in control condition** | **Number of regions**  **in E2-treated condition** |
| --- | --- | --- |
| 1% | 1448 | 1447 |
| 5% | 1436 | 1434 |
| 10% | 1412 | 1372 |
| 20% | 923 | 506 |
| 30% | 177 | 84 |
| 40% | 45 | 27 |
| 50% | 18 | 9 |
| 60% | 8 | 5 |

**Table S2**. Functional annotation of genes located in the top 50 cold (500 genes) and the top 50 hot (280 genes) chromosomal interaction regions by using DAVID. The top 10 of each functional annotation are presented at here.

|  | | GO Term | Tissue expression | Disease | Pathways |
| --- | --- | --- | --- | --- | --- |
| Top 50 cold regions | Arylsulfatase activity (5 genes).  Sulfuric ester hydrolase activity (5 genes).  Phosphatidylcholine biosynthetic process (4 genes).  Phosphatidylcholine metabolic process (4 genes).  Microtubule nucleation (3 genes).  Ethanolamine and derivative metabolic process (4 genes).  Biogenic amine metabolic process (6 genes).  Clathrin-coated vesicle (7 genes).  Beta-amyloid binding (3 genes).  Cytoplasmic vesicle (18 genes). | Uncharacterized tissue uncharacterized histology^3rd^ (14 genes).  Pancreas normal^3rd^ (95 genes).  Pancreatic tumor disease^3rd^ (84 genes).  Salivarygland^3rd^ (165 genes).  Tonsil^3rd^ (148 genes).  Testis Germ Cell^3rd^ (59 genes).  Mammary gland breast carcinoma cell line^3rd^ (52 genes).  26786:uncharacterized tissue uncharacterized histology^3rd^ (5 genes).  Pancreatic islet normal3^rd^ (12 genes).  28202:uncharacterized tissue uncharacterized histology^3rd^ (3 genes). | Beckwith-Wiedemann syndrome (3 genes).  Schizophrenia (11 genes).  Skin/hair/eye pigmentation 1, blue/nonblue eyes (2 genes).  Skin/hair/eye pigmentation 1, blond/brown hair (2 genes).  Beta-cell function; insulin resistance (2 genes).  Body mass; triglycerides; blood pressure, arterial (2 genes).  Sarcoma, synovial (2 genes).  Alcoholism (4 genes).  Schizotypal traits (2 genes).  PSYCH (15 genes). | 3.1.6.- (4 genes).  TGF-beta signaling pathway (7 genes).  3.1.6.1 (2 genes).  Pyrimidine metabolism (5 genes).  Alzheimer disease-amyloid secretase pathway (4 genes).  Interferon-gamma signaling pathway (3 genes).  hsa04670:Leukocyte transendothelial migration (5 genes).  FGF signaling pathway (5 genes). |  |
| Top 50 hot regions | Intracellular signaling cascade (35 genes).  Heterotrimeric G-protein complex (5 genes).  Aryldialkylphosphatase activit (3 genes).  Arylesterase activity (3 genes).  Vesicle-mediated transport (18 genes).  Golgi apparatus part (12 genes).  FAD binding (6 genes).  Purine nucleotide binding (38 genes).  Regulation of osteoblast differentiation (5 genes).  Intracellular receptor-mediated signaling pathway (6 genes). | 77:Mammary gland carcinoma^3rd^ (40 genes).  BM-CD105+Endothelial^3rd^ (80 genes).  Amygdala^3rd^ (63 genes).  79:Mammary gland carcinoma^3rd^ (39 genes).  78:Mammary gland carcinoma^3rd^ (39 genes).  Bone marrow^3rd^ (48 genes).  Mammary gland normal ^3rd^ (77 genes).  TONGUE^3rd^ (67 genes).  39035:mammary gland neoplasia^3rd^ (11 genes).  TemporalLobe^3rd^ (59 genes). | Pseudohypoparathyroidism, type Ib (3 genes).  Atherosclerosis, coronary; diabetes, type 2; lipids; stroke, ischemic (3 genes).  ALS/amyotrophic lateral sclerosis (4 genes).  CANCER (21 genes).  Many sequence variants affecting diversity of adult human height (7 genes).  Hearing loss/deafness (3 genes).  Breast cancer (10 genes).  Paraoxonase (2 genes).  Clonal homozygosity of rectal cell carcinoma (2 genes).  Atherosclerosis, coronary; hypercholesterolemia (2 genes). | 3.1.8.1 (3genes).  1.16.1.- (3 genes).  P00048:PI3 kinase pathway ( 7 genes).  P00040:Metabotropic glutamate receptor group II pathway (5 genes).  P00043:Muscarinic acetylcholine receptor 2 and 4 signaling pathway ( 5 genes).  P05731:GABA-B_receptor_II_signaling (4 genes).  hsa04512:ECM-receptor interaction (5 genes).  hsa05222:Small cell lung cancer (5 genes).  P04373:5HT1 type receptor mediated signaling pathway (4 genes).  P00026:Heterotrimeric G-protein signaling pathway-Gi alpha and Gs alpha mediated pathway (7 genes). |  |

**Table S3**. Number of strong chromosomal interaction changes between the E2-treated and control conditions (1Mb resolution, absolute relative ratio equals to 2 and the Z-score greater than and equals to 1).

|  | Num. of gain-interactions in E2-treated condition | Num. of loss-interactions at E2-treated condition |
| --- | --- | --- |
| Intra-chromosomal | 3,194 | 1,114 |
| Inter-chromosomal | 9,134 | 13,786 |
| Total interaction | 12,328 | 14,900 |

**Table S4**. Number of general chromosomal interaction changes between E2-treated and control conditions. (e.g., 1Mb resolution, absolute relative ratio >=0.67 and Z-score not equal to 0)

|  | Num. of gain-interactions at E2-treated, relative ratio>=0.67 | Num. of loss-interactions at E2-treated, relative ratio<=-0.67 |
| --- | --- | --- |
| Intra-chromosomal | 29,682 | 17,452 |
| Inter-chromosomal | 187,873 | 241,554 |
| Total interaction | 217,555 | 259,006 |

**Table S5**. Top 10 chromosomal regions (1 Mb resolution) with the most lost interactions and the most gained interactions, respectively. Based on four types of the strongest interaction changes (Additional file 6), we counted for each region (1Mb resolution) that how many interactions are gained (positive value) and how many are lost after the E2 treatment (negative value). If the region is also appeared in the top 10 hot interaction regions (Table 2) then it is colored by red.

| Chr | Start | End | Num. of loss-interactions | Num. of gain-interactions |
| --- | --- | --- | --- | --- |
| 20 | 52000001 | 53000000 | -253 | 170 |
| 20 | 51000001 | 52000000 | -212 | 137 |
| 20 | 45000001 | 46000000 | -167 | 101 |
| 17 | 56000001 | 57000000 | -164 | 96 |
| 17 | 57000001 | 58000000 | -158 | 108 |
| 17 | 55000001 | 56000000 | -138 | 107 |
| 17 | 54000001 | 55000000 | -123 | 78 |
| 20 | 55000001 | 56000000 | -78 | 44 |
| 20 | 46000001 | 47000000 | -62 | 40 |
| 20 | 56000001 | 57000000 | -46 |  |
| 20 | 53000001 | 54000000 |  | 36 |

**Table S6**. Correlation matrix of different replicates of Hi-C data at different time points.

| Before E2 treatment | | | | |
| --- | --- | --- | --- | --- |
| Replicates | 1 | 2 | 3 | 4 |
| 1 | 1 | 0.9414 | 0.9396 | 0.9244 |
| 2 | 0.9414 | 1 | 0.9429 | 0.9269 |
| 3 | 0.9396 | 0.9429 | 1 | 0.9271 |
| 4 | 0.9244 | 0.9269 | 0.9271 | 1 |
|  |  |  |  |  |
|  |  |  |  |  |
|  |  |  |  |  |
| After E2 treatment | | | | |
| Replicates | 1 | 2 | 3 | 4 |
| 1 | 1 | 0.9507 | 0.9424 | 0.9395 |
| 2 | 0.9507 | 1 | 0.9462 | 0.9434 |
| 3 | 0.9424 | 0.9462 | 1 | 0.9579 |
| 4 | 0.9395 | 0.9434 | 0.9579 | 1 |

**Supplementary Methods**

**ChIP-seq analysis**

BALM (Lan, et al., 2011) program is used to analyze ChIP-seq data in this study for its high resolution in detecting peaks. Briefly, the signal tags produced by ChIP-seq are modeled as a mixture of Bi-Asymmetric Laplace distribution. Next, expectation maximization (EM) algorithm is applied to separate the components (close positioned peaks) of the mixture model. Finally, the best mixture model is chosen using the Bayesian Information Criterion (BIC).

**Defining interacting loci**

In addition to the biases presented in regular sequencing data such as unequal efficiency of DNA amplification, copy number differences, existence of amplicon, sequencing bias, image processing and matching errors, in Hi-C experiments, self ligation and random ligation also give rise to false positive. Self ligated loop forms when the two ends of a single enzyme cut DNA fragment ligated hence prevent it from being digested by exonuclease. Random ligation is formed by two or multiple random floating DNA fragments. In this study, a latent class Poisson regression model (Yang and Lai, 2004) and a filter pipeline were built to control false positives and classify sequenced DNA fragments. We define proximate ligation event as a ligation between two ends that are spatially adjacent to each other. Both self ligation and ligation between two ends of closely positioned chromatin fragments are in this category.

*Latent class Poisson regression model* We model the proximate ligation event and random ligation event as two independent Poisson distribution and thus, the overall ligation event could be represented by latent class model with two hidden variable*.* Hence, the probability that $Y_{i}$ is from a particular class *k* is given by


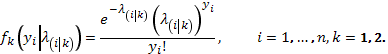


Where ${??}_{i|k}$ is the mean rate of individual *i* given that it is in class *k* and n denotes the total number is ligation event.

The canonical log link function used to transform the mean of the Poisson distribution to linear predictor ***β_k_*** is


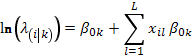


Where are the explanatory variables.


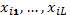

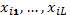


Expectation Maximization (EM) algorithm^1, 2, 3^ is applied to estimate the unknown parameter ***β_k_*** as well as which is the proportion of *k*th class in all ligation event with , .


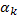

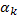

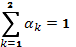

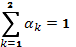

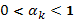

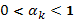


False discovery rate (FDR) is defined as the proportion of proximate ligation in total identified ligation event. Given a threshold enrichment of hybrid fragments *t*, the *FDR* could be calculated by the following formula,


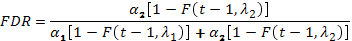


Where $F(t-1,??)$ is the cumulative distribution function of Poisson distribution.

In this study, we set *t* = 2 (*FDR* = 8.35%) with the consideration of both sensitivity and specificity.

*Determine interacting loci* The above model was not able to eliminate self ligation since the two ends of a single DNA fragment also possess spatial proximity. To achieve this objective and further classify proximate ligation events, a filter was applied. Briefly, a hybrid fragment with two ends mapped to different chromosomes was defined as an inter-chromosomal hybrid fragments. If the coordinates of the forward strand end on hg18 is larger than that of the reward strand end and the distance between these two ends is less than 20kb, the hybrid fragment was considered as a self ligated loop. Otherwise, a hybrid fragment with both ends aligned to the same chromosome and not self ligated was classified to intra-chromosomal hybrid fragment. If the number of hybrid fragments indicating an interaction between two loci exceed the threshold, *t*, these two loci were defined as interactive loci.

**Correlation between replicates**

Genome was divided into 1000bp bins and the numbers of reads
of each bin for 4 different replicates were counted. Then a random sample of 30,227
bins is used to to calculate the correlation matrix between each pair of replicates (**Figure S16, Table S6**).

**Supplementary References**

Hsu, P.Y., Hsu, H.K., Singer, G.A., Yan, P.S., Rodriguez, B.A., Liu, J.C., Weng, Y.I., Deatherage, D.E., Chen, Z., Pereira, J.S., Lopez, R., Russo, J., Wang, Q., Lamartiniere, C.A., Nephew, K.P. and Huang, T.H. (2010) Estrogen-mediated epigenetic repression of large chromosomal regions through DNA looping, *Genome research*, **20**, 733-744.

Lan, X., Adams, C., Landers, M., Dudas, M., Krissinger, D., Marnellos, G., Bonneville, R., Xu, M., Wang, J., Huang, T.H., Meredith, G. and Jin, V.X. (2011) High resolution detection and analysis of CpG dinucleotides methylation using MBD-Seq technology, *PloS one*, **6**, e22226.

Lieberman-Aiden, E., van Berkum, N.L., Williams, L., Imakaev, M., Ragoczy, T., Telling, A., Amit, I., Lajoie, B.R., Sabo, P.J., Dorschner, M.O., Sandstrom, R., Bernstein, B., Bender, M.A., Groudine, M., Gnirke, A., Stamatoyannopoulos, J., Mirny, L.A., Lander, E.S. and Dekker, J. (2009) Comprehensive mapping of long-range interactions reveals folding principles of the human genome, *Science (New York, N.Y*, **326**, 289-293.

Yang, M. and Lai, C. (2004) Mixture poisson regression models for heterogeneous count data based on latent and fuzzy class analysis, *Soft computing*, 519-524.
